# Comparative chloroplast genome analyses of *Paraboea* (Gesneriaceae): Insights into adaptive evolution and phylogenetic analysis

**DOI:** 10.3389/fpls.2022.1019831

**Published:** 2022-10-05

**Authors:** Yifei Wang, Fang Wen, Xin Hong, Zhenglong Li, Yaolei Mi, Bo Zhao

**Affiliations:** ^1^ Department of Pharmacognosy, Guilin Medical University, Guilin, China; ^2^ Department of Pharmacy, Guilin Medical University, Guilin, China; ^3^ Guangxi Key Laboratory of Plant Conservation and Restoration Ecology in Karst Terrain, Guangxi Institute of Botany, Guangxi Zhuang Autonomous Region and Chinese Academy of Sciences, Guilin, China; ^4^ Anhui Provincial Engineering Laboratory of Wetland Ecosystem Protection and Restoration, School of Resources and Environmental Engineering, Anhui University, Hefei, China; ^5^ Yunnan Key Laboratory for Integrative Conservation of Plant Species with Extremely Small Populations, Kunming Institute of Botany, Chinese Academy of Sciences, Kunming, China; ^6^ Institute of Chinese Materia Medica, China Academy of Chinese Medical Sciences, Beijing, China

**Keywords:** *Paraboea*, phylogenetic, positive selection, chloroplast, genome

## Abstract

*Paraboea* (Gesneriaceae) distributed in the karst areas of South and Southwest China and Southeast Asia, is an ideal genus to study the phylogeny and adaptive evolution of karst plants. In this study, the complete chloroplast genomes of twelve *Paraboea* species were sequenced and analyzed. Twelve chloroplast genomes ranged in size from 153166 to 154245 bp. Each chloroplast genome had a typical quartile structure, and relatively conserved type and number of gene components, including 131 genes which are composed of 87 protein coding genes, 36 transfer RNAs and 8 ribosomal RNAs. A total of 600 simple sequence repeats and 389 non-overlapped sequence repeats were obtained from the twelve *Paraboea* chloroplast genomes. We found ten divergent regions (*trn*H-GUG-*psb*A, *trn*M-CAU, *trn*C-GCA, *atp*F-*atp*H, *ycf1*, *trn*K-UUU-*rps*16, *rps*15, *pet*L, *trn*S-GCU-*trn*R-UCU and *psa*J-*rpl*33) among the 12 *Paraboea* species to be potential molecular markers. In the phylogenetic tree of 31 Gesneriaceae plants including twelve *Paraboea* species, all *Paraboea* species clustered in a clade and confirmed the monophyly of *Paraboea*. Nine genes with positive selection sites were detected, most of which were related to photosynthesis and protein synthesis, and might played crucial roles in the adaptability of *Paraboea* to diverse karst environments. These findings are valuable for further study of the phylogeny and karst adaptability of Gesneriaceae plants.

## Introduction

Limestone areas have diverse and unique regional microhabitat and special island habitats (Xin et al., 2021). These special habitats (such as stone mountains, karst caves, Tiankeng, etc.) provide very favorable conditions for species isolation and differentiation. After a long period of evolution and natural selection, limestone areas have bred a high degree of species diversity and significant endemism. With the development of the economy, due to frequent human activities (such as tourism development, etc.), the fragile ecological balance of these diversified microhabitats is easily destroyed, and the survival of karst plants is threatened. Studying the adaptive evolution of karst plants has extremely important practical significance for the protection of karst plants (Tao et al., 2016).

*Paraboea*, an important genus of Gesneriaceae, contains about 144 species (Xu et al., 2012; Wen et al., 2013; Puglisi and Phutthai, 2017). The genus is mainly distributed in karst areas of south China, southwest China and southeast Asia, China has about 29 species, of which 19 are endemic (Guo et al., 2016). Among these species, some are widely distributed and attached to the rock gaps of stone mountains in direct sunlight (such as *P. rufescens* and *P. swinhoei*), some are distributed in dark and wet caves (such as *P. filipes*), and some grow on limestone rocks or inter stone soil in the dark place under the dense forest from the hillside to the top of the mountain (such as *P. barbatipes*) (Zhou et al., 2003; Gao et al., 2006). Considering the distribution of *Paraboea* species in diverse microhabitats, it becomes an excellent group to study the adaptive evolution of karst plants. However, the phylogenetic study of *Paraboea* is mainly based on a small number of chloroplast or nuclear gene markers, and the phylogenetic relationship has not been completely solved, which greatly limits the discussion of adaptive evolution of genes and traits. Based on nuclear ITS (internal transcribed spacer) sequences and chloroplast genome sequences (*trn*H-*psb*A spacer), the phylogenetic relationship of *Paraboea* was reconstructed, and the taxonomic boundaries among some related species were clarified (Li and Wang, 2007; Puglisi et al., 2011; Xin et al., 2019; Guo et al., 2020). However, the existing chloroplast genome sequences cannot completely solve the phylogenetic relationship of *Paraboea*. It is necessary to add faster and more suitable molecular sequences to reconstruct its phylogenetic relationship.

The chloroplast genome is one of the important molecular tools to study plant adaptive evolution. The challenging environment may impose selective pressure on genes related to photosynthesis, leaving the footprints of natural selection on genes. The main protein coding genes of the chloroplast genome include those controlling genetic and photosynthetic systems as well as genes encoding other functions. Photosynthetic system genes are genes related to photosynthesis, which are responsible for encoding members of ATP synthase, Rubisco large subunit, NADPH dehydrogenase and photosystem I and II (Zhang et al., 2018). The adaptive evolution analysis showed that chloroplast genes related to photosynthesis generally had positive selection sites in plants living in various extreme environments, and these gene regions might play a crucial role in plant adaptation to different environments (Chen et al., 2021).

The chloroplast genome sequences not only provide full-length protein coding sequences for the adaptive evolution of genes related to photosynthesis under the selection pressure of different environments, but also screen suitable hypervariable regions to solve the phylogenetic relationship of plants (Yang et al., 2020). The size of chloroplast genome in terrestrial plants is 120-160 kb, encoding 110-130 unique genes. Because of the slow evolutionary rate of change, maternal inheritance, less recombination and satisfactory collinearity between the sequences of various plant groups, the chloroplast genome sequences were suitable for molecular markers (Zhai et al., 2021). With the development of Next-generation sequencing technology, a large number of chloroplast genome data can be easily obtained. Based on chloroplast comparative genomics analyses, the high variation regions were located to develop specific molecular markers of groups or species for applying to the research of phylogenetic analysis and species identification (Chen et al., 2022; Song et al., 2022).

So far, there was no scientific research related to the complete chloroplast genome of *Paraboea*. In this study, we sequenced, assembled and analyzed the chloroplast genomes of twelve *Paraboea* species, and constructed the phylogenetic relationship of 31 species belonging to 12 genera of Gesneriaceae based on protein coding sequences. We also calculated selective pressures to investigate whether the coding protein genes in *Paraboea* species were under purifying selection or positive selection. Comprehensive insights into the character and evolution of the chloroplast genomes, provided a theoretical basis for the protection and rational utilization of germplasm resources of *Paraboea* plants in karst areas.

## Materials and methods

### Plant materials and DNA extraction

The 12 species of *Paraboea* in China and Vietnam used in the study were identified, collected and finally cultivated in the Guangxi Institute of Botany (**Table 1**). Fresh green leaves were sampled, washed, dried and stored at -80°C till DNA extraction (Feng et al., 2020). The total genomic DNA was extracted according to the modified CTAB method (Doyle and Doyle, 1987).

**Table 1:** Sources of material from twelve *Paraboea* species.

Taxon	Voucher	GenBank accession number	Location	Habitat
*P. clavisepala*	ZBPC202100061	MZ465381	Jingxi Guangxi, China	Limestone; ca. 800 m
*P. dictyoneura*	ZBPD202100062	MZ465383	Yingde Guangdong, China	Rocks in forests; 100-800 m
*P. dolomitica*	ZBPD202100063	MZ465376	Shibing Guizhou, China	rock faces of dolomite karst area, ca. 650-855 m
*P. filipes*	ZBPF202100064	MZ465379	Lianzhou Guangdong, China	Limestone cliffs; ca.100-300 m
*P. glutinosa*	ZBPG202100065	MZ465382	Caobang, Vietnam	Rocks of slopes; ca. 400-1400 m
*P. guilinensis*	ZBPG202100066	MZ465377	Guilin Guangxi, China	Limestone cliffs
*P. martinii*	ZBPM202100067	MZ465385	Napo Guangxi, China	limestone under the hillside forest; ca. 1220-1260 m
*P. peltifolia*	ZBPP202100068	MZ465386	Mashan Guangxi, China	Limestone; ca. 300-400 m
*P. rufescens*	ZBPR202100069	MZ465384	Napo Guangxi, China	On rocks of limestone hills and valley forests; ca. 200-1500 m
*P. sinensis*	ZBPS202100070	MZ465380	Longzhou Guangxi, China	Crevices of rocks or on cliffs in forests; ca. 600-2500 m
*P. swinhoei*	ZBPS202100071	MZ465378	Rongshui Guangxi, China	Shady and damp rocks under forests; ca. 300-1000 m
*P. wenshanensis*	ZBPW202100072	MZ465375	Wenshan Yunnan, China	moist shady cliffs of limestone hills, ca. 1500 m

### Genome sequencing and assembling

Qualified DNA fragments were obtained by mechanical fracture method, and were sequenced after purification, terminal repair and other processing. The 350 bp fragment was screened by agarose gel electrophoresis and amplified by PCR to construct a sequence library. Paired-end (PE) reads were obtained using the Illumina HiSeq 2000 sequencer (Illumina Biotechnology Company, San Diego, CA, USA) (Gu et al., 2018). *De novo* genome assembly from the clean data was accomplished utilizing NOVOPlasty v2.7.2 (Dierckxsens et al., 2017), with a k-mer length of 39 bp and the chloroplast genome of *Primulina huaijiensis* (NC_036413) as the reference sequence. The correctness of the assembly was confirmed by manually editing and mapping all the raw reads to the assembled genome sequence using Bowtie2 (v2.0.1) (Langmead et al., 2009) under the default settings. Finally, the complete chloroplast genome sequences of twelve *Paraboea* species were obtained.

### Genome annotation and sequence characterization

Functional annotation of the chloroplast genome includes coding gene prediction and non-coding RNA (rRNA and tRNA) annotation. Using CPGAVAS2 (Shi et al., 2019), the twelve complete chloroplast genomes were annotated with a reference genome (*Primulina huaijiensis*, GenBank: NC036413). Meanwhile, tRNA scan-SE version 1.21 (Schattner et al., 2005) was used to identify and confirm tRNA genes. The twelve circular chloroplast genome maps were constructed using the OrganellarGenomeDRAW (OGDRAW) v.1.3.1 tool followed by manual modification (Greiner et al., 2019). And the whole twelve sequences were submitted to GenBank (**Table 1**).

### Repeat sequences and SSR analysis

The Perl script MISA (http://pgrc.ipk-gatersleben.de/misa/) (Beier et al., 2017) was used with the filter thresholds set to detect SSRs. The specific parameters were set at repeat units ≥ 8 for mononucleotides, repeat units ≥ 4 for dinucleotides and trinucleotides, and repeat units ≥ 3 for tetranucleotides, pentanucleotides and hexanucleotides. To identify complex repeative sequences such as forward, reverse, complement and palindromic, REPuter online software (Kurtz et al., 2001) was used with a minimum repeat size of 30 bp and 90% sequence identity (Hamming distance of 3).

### Boundary regions and genome comparative analysis

In order to better display the expansion/contraction events of the IR region, the connecting regions of IR-LSC and IR-SSC in the chloroplast genomes of twelve *Paraboea* species were compared by using IRscope (https://irscope.shinyapps.io/irapp/) online software (Amiryousefi et al., 2018). To identify interspecific variations, the mVISTA online software was used to compare the chloroplast genomics of twelve *Paraboea* plants (Frazer et al., 2004). The comparative analysis was carried out by using the shuffle-LAGAN mode in mVISTA with the annotation of *P. sinensis* as reference, and the sequence alignment was visualized in an mVISTA plot. We used MEGA v6.0 (Tamura et al., 2013) to calculate the percentage of variable sites in the protein-coding genes. We also used DnaSP v6.0 (Rozas et al., 2017) to calculate the nucleotide polymorphism (Pi) among the twelve *Paraboea* species. When calculating the Pi value, set the windows length to 100 sites and the step size to 25 sites.

### Phylogenetic analysis

The complete chloroplast protein-coding genes of 31 Gesneriaceae species (12 *Paraboea* species in this study and 20 other species from NCBI) were aligned using MUSCLE v3.8.31 (Edgar, 2004), and then aligned in MAFFT (version 7.222) using the default parameters (Kazutaka and Standley, 2013). The final two sequence alignment results are consistent. The aligned sequences were used to construct the phylogenetic trees using the maximum likelihood (ML) method implemented in RAxML 7.0.4 (Stamatakis, 2006) with 1000 replicates under the GTR + CAT model.

### Adaptive evolution analysis

In order to detect the positive selection of chloroplast genes in *Paraboea*, the non-synonymous (DN) and synonymous (DS) substitution rates of protein-coding genes and the DN/DS (ω) values of protein-coding genes were calculated. All of the CDS sequences were extracted from chloroplast genome, and then the single-copy CDS sequences common to all species were selected and aligned with the codon model. We used EasyCodeML v1.21 (Gao et al., 2019b) to identified positive selection sites. A total of 76 CDSs presented in all the analysed species, and were used for identification of positive selection using the site model (seqtype = 1, model = 0, NSsites = 0, 1, 2, 3, 7, 8). In addition, Bayes Empirical Bayes (BEB) method (Huelsenbeck and Ronquist, 2001) was used to calculate the posterior probabilities for amino acid sites that were potentially under positive selection. The results showed that the amino acid sites with a posteriori probability of more than 0.95 were positive selected. Moreover, the logarithmic likelihood value of site models was calculated by likelihood ratio test (LRT) and its statistical significance. Finally, we used the PSIPRED server (Buchan et al., 2013) to visualize the amino acid sequences of positively selected gene secondary structure, and used the SWISS-MODEL online software (Waterhouse et al., 2018) to predict the protein structure of these genes.

## Results

### General features of chloroplast genomes

**Figure 1:**
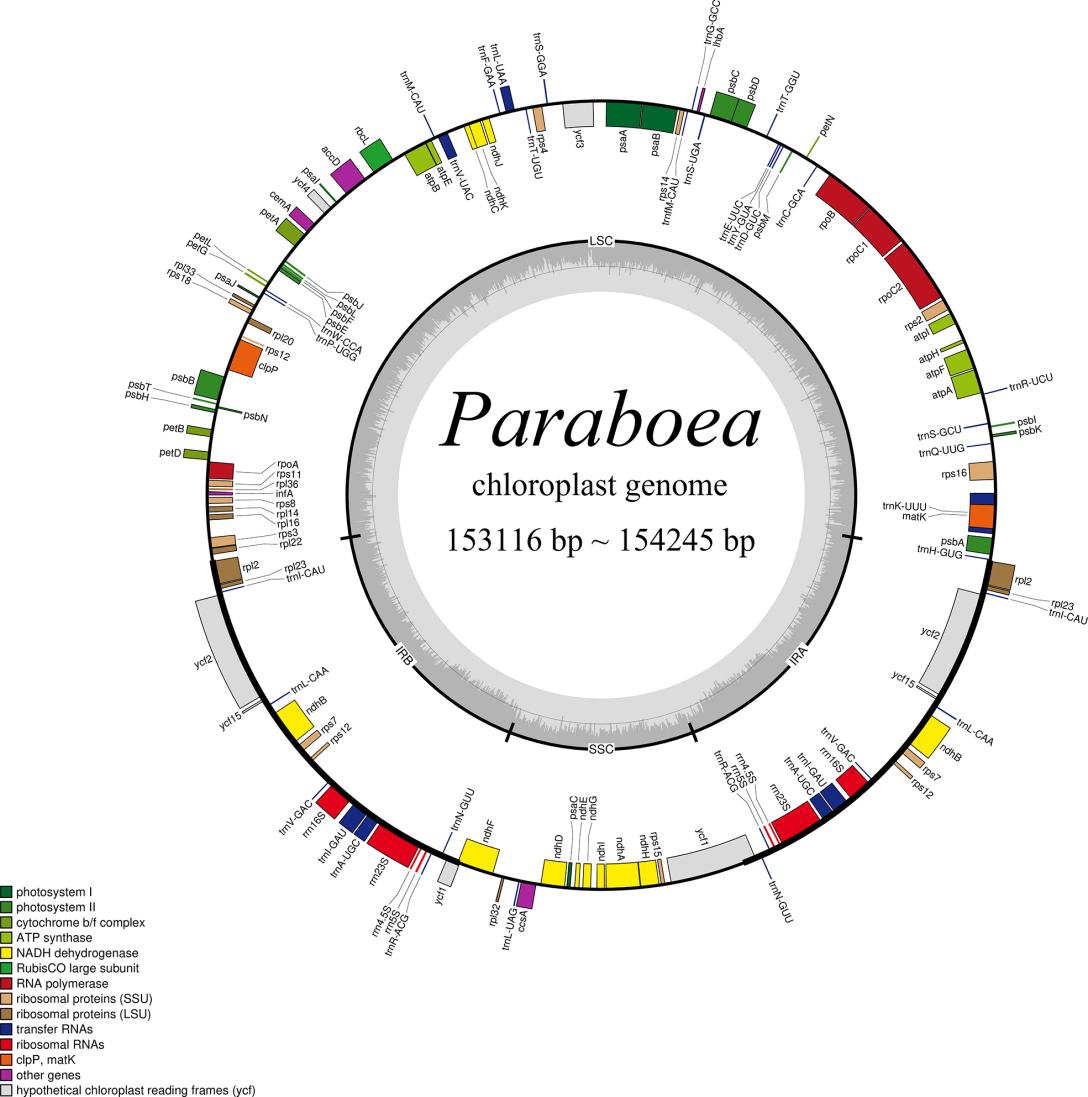
Structural map of the *Paraboea* chloroplast genome. Genes shown outside the outer circle are transcribed clockwise and those inside are transcribed counterclockwise. Genes belonging to different functional groups are color-coded. *P. clavisepala* is used as the template for **Figure 1**.

In this study, the chloroplast genomes of twelve *Paraboea* species were sequenced and characterized. Each chloroplast genome was made up of three distinct regions: a small single copy region (SSC), a large single copy region (LSC) and two inverted repeat regions (IRs) (**Figure 1**). The complete chloroplast genomes of the 12 *Paraboea* species ranged from 153166 bp (*P. guilinensis*) to 154245 bp (*P. wenshanensis*) in length (**Table 2**). The length of SSC ranged from 17656 bp (*P. glutinosa*) to 18089 bp (*P. wenshanensis*), while the length of LSC and IR length ranged from 84761 bp (*P. clavisepala*) to 85488 bp (*P. wenshanensis*), and from 25272 bp (*P. dolomitica*) to 25334 bp (*P. wenshanensis*). In all twelve *Paraboea* species, the chloroplast genomes of *P. filipes* and *P. wenshanensis* had the lowest total GC content (37.45%), while the chloroplast genome of *P. martini*had the highest total GC content (37.72%). Gene annotation showed that each chloroplast genome contained 131 genes in conserved order and orientation, which contained 8 ribosomal RNA (rRNA) genes, 36 transfer RNAs (tRNAs), and 87 protein-coding genes (**Table 3**). Fifteen genes (10 protein coding genes and 5 tRNA genes) with introns were identified. Among them, the *clp*P and *ycf3* genes had two introns, respectively, while the other 13 genes had one intron.

**Table 2:** Summary of the chloroplast genomes of twelve *Paraboea* species.

	Genome Length (bp)	LSC Length (bp)	SSC Length (bp)	IR Length (bp)	GC (%)	Total Genes	CDS	tRNA	rRNA
*P. clavisepala*	153398	84761	18045	25296	37.58%	131	87	36	8
*P. dictyoneura*	153406	84829	17999	25289	37.52%	131	87	36	8
*P. dolomitica*	153510	84885	18081	25272	37.45%	131	87	36	8
*P. filipes*	153486	84851	18001	25317	37.45%	131	87	36	8
*P. glutinosa*	153505	85303	17656	25273	37.66%	131	87	36	8
*P. guilinensis*	153166	84819	17759	25294	37.57%	131	87	36	8
*P. martinii*	153580	84978	18032	25285	37.72%	131	87	36	8
*P. peltifolia*	153459	84784	18043	25316	37.55%	131	87	36	8
*P. rufescens*	153352	85098	17694	25280	37.69%	131	87	36	8
*P. sinensis*	153453	84869	18028	25278	37.61%	131	87	36	8
*P. swinhoei*	153564	85160	17800	25302	37.65%	131	87	36	8
*P. wenshanensis*	154245	85488	18089	25334	37.70%	131	87	36	8

LSC, large single-copy; SSC, small single-copy; IR, inverted repeat.

**Table 3:** Genes in the chloroplast genome of twelve *Paraboea* species.

Category	Gene group	Gene name
Protein synthesis and DNA- replication	Ribosomal RNA genes	*rrn4.5, rrn5, rrn16, rrn23*
	Transfer RNA genes	trnA-UGC^*^, trnC-GCA, trnD-GUC, trnE-UUC, trnF-GAA, trnG-GCC, trnH-GUG, trnI-CAU, trnI-GAU^*^, trnK-UUU^*^, trnL-CAA, trnL-UAA^*^, trnL-UAG, trnM-CAU, trnfM-CAU, trnN-GUU, trnP-UGG, trnQ-UUG, trnR-UCU, trnR-ACG, trnS-GCU, trnS-GGA, trnS-UGA, trnT-GGU, trnT-UGU, trnV-GAC, trnV-UAC^*^, trnW-CCA, trnY-GUA
	Ribosomal protein genes (larger subunit)	rpl2^*^, rpl14, rpl16^*^, rpl20, rpl22, rpl23, rpl32, rpl33, rpl36
	Ribosomal protein genes (smaller subunit)	*rps2, rps3, rps4, rps7, rps8, rps11, rps12, rps14, rps15, rps16, rps18*
	RNA polymerase	rpoA, rpoB, rpoC1^*^, rpoC2
Photosynthesis	Photosystem I	*psaA, psaB, psaC, psaI, psaJ*
	Photosystem II	*psbA, psbB, psbC, psbD, psbE, psbF, psbH, psbI, psbJ, psbK, psbL, psbM, psbN, psbT*
	Cytochrome b/f complex	*petA, petB, petD, petG, petL, petN*
	ATP synthase	atpA, atpB, atpE, atpF^*^, atpH, atpI
	Rubisco large subunit	*rbcL*
	NADH dehydrogenase	ndhA^*^, ndhB^*^, ndhC, ndhD, ndhE, ndhF^*^, ndhG, ndhH, ndhI, ndhJ, ndhK
Miscellaneous group	ATP-dependent protease	clpP^**^
	Maturase	*matK*
	Acetyl-CoA carboxylase	*accD*
	Cytochrome c biogenesis	*ccsA*
	Inner membrane protein	*cemA*
Pseudogene unknown function	Hypothetical chloroplast reading frames (ycf)	ycf1^*^, ycf2, ycf3^**^, ycf4, ycf15
Other gene	LhbA	*lhbA*

“*” indicates the presence of one intron.

“**” indicates the presence of two introns.

### IR expansion and contraction in the twelve *Paraboea* chloroplast genomes

**Figure 2:**
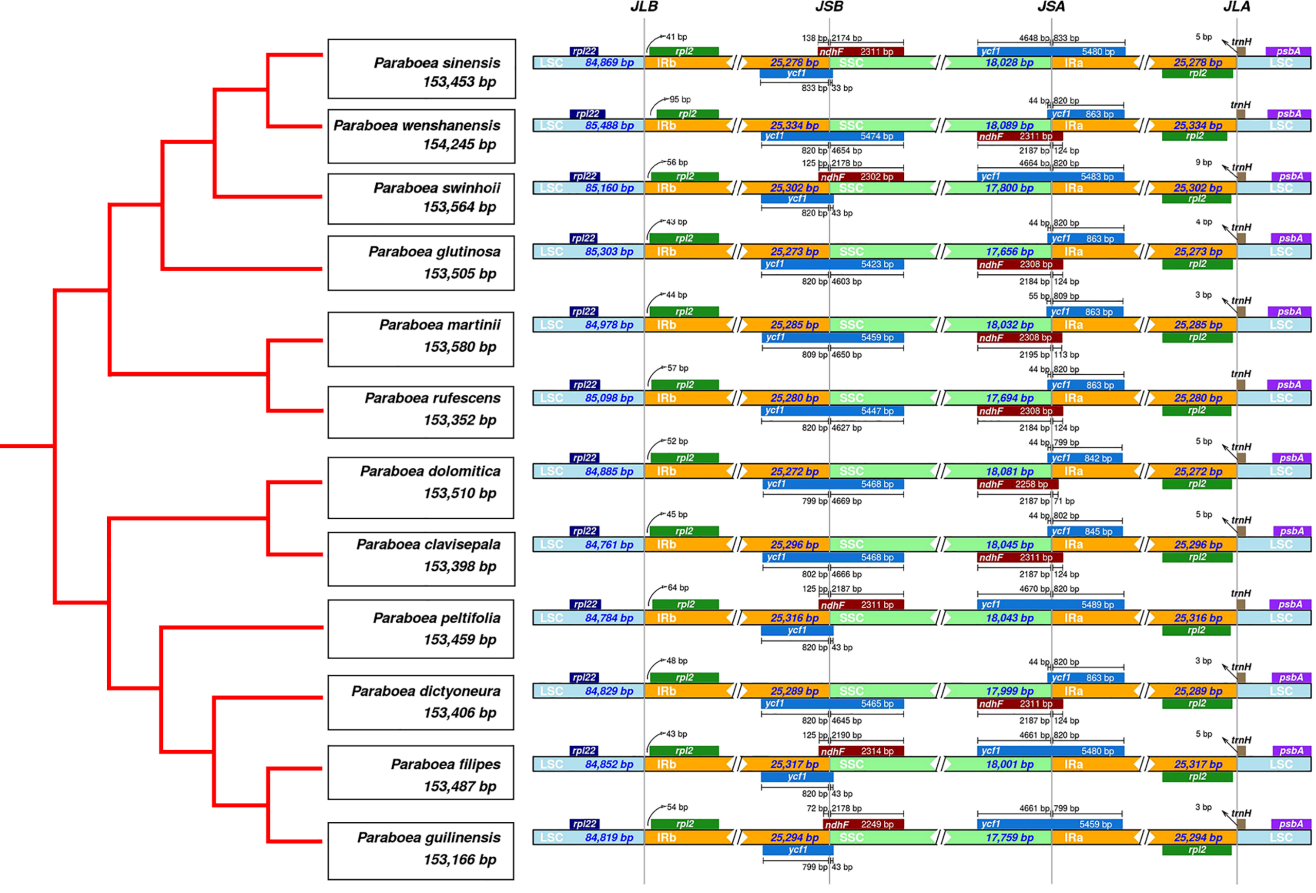
Comparison of the borders of the LSC, SSC, and IR regions among twelve chloroplast genomes.

There were 4 borders between LSC, IRb, IRa and SSC in the cpGenome: LSC/IRb border (JLB line), IRb/SSC border (JSB line), SSC/IRa border (JSA line), IRa/LSC border (JLA line). The borders of the twelve *Paraboea* chloroplast genomes were compared (**Figure 2**). The LSC/IRb border and IRa/LSC border were relatively conservative. The *rpl2* gene located at the LSC/IR border, and the distances between *rps2* and the JLB line ranged from 41 bp to 95 bp. The *trn*H-GUG noncoding gene located on the right side of the JLA line with a distance of 0 to 9 bp.

At the IRb/SSC border, the *ndh*F encoding gene located at the IRB-SSC boundary. In the chloroplast genome of *P. sinensis*, *P. swinhoei*, *P. peltifolia*, *P. filipes* and *P. guilinensis*, the *ndh*F gene had the length of 72 bp (*P. guilinensis*) to 138 bp (*P. sinensis*) in the IRB region. In the other seven *Paraboea* chloroplast genomes, the *ndh*F gene spanned the IRb/SSC border and had the length of 113 bp to 124 bp.

At the SSC/IRa border, the *ycf1* gene spanned the SSC-IRB boundary. Due to the special position of the *ycf1* gene, there were seven *Paraboea* chloroplast genomes in the IRA region with *ycf1* pseudogenes, the corresponding length ranged from 842 bp to 863 bp in the IRA region. And in the other *Paraboea* species chloroplast genomes, the *ycf1* gene was located on the SSC-IRA boundary, which made the corresponding pseudogene take place in the IRB region with the length of 799 bp to 833 bp.

### Repeat sequence analysis

**Figure 3:**
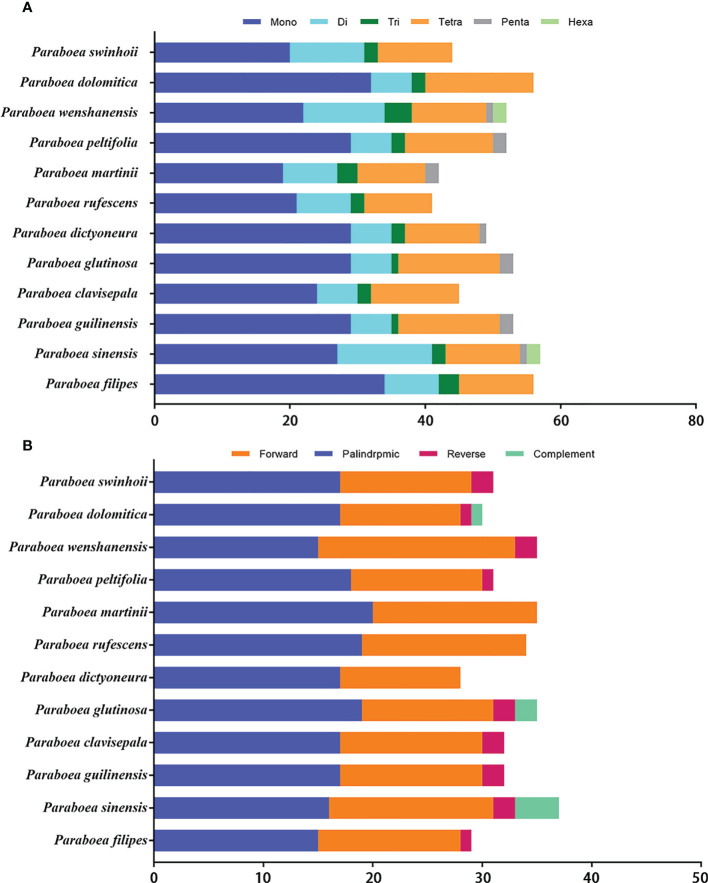
The distribution maps of sequence repeats. **(A)** Number of different types of SSRs present in twelve *Paraboea* chloroplast genomes. **(B)** The comparison of the four complex repeat types among twelve *Paraboea* choloplast genomes.

The twelve *Paraboea* chloroplast genomes contained 600 SSRs (**Figure 3A** and **Supplementary Table S2**). In the chloroplast genome of *P. rufescens*, 41 SSRs were detected, which was the least of the 12 chloroplast genomes. And in the chloroplast genome of *P. sinensis*, a total of 57 SSRs were identified, which was the most of the 12 chloroplast genomes. For each *Paraboea* specie, mononucleotide repeats were the most common, with numbers ranging from 19 to 34; followed by tetranucleotides ranging from 10 to 16; dinucleotides ranging from 6 to 14; trinucleotides ranging from 1 to 4; pentanucleotides ranging from 0 to 2 and hexanucleotide ranging from 0 to 2 (**Supplementary Figure S1**).

Non-overlapped sequence repeats including forward repeats, reverse repeats, palindromic repeats and complement repeats were detected in twelve chloroplast genomes. A total of 389 non-overlapped sequence repeats were detected in twelve chloroplast genomes of *Paraboea* plants (**Figure 3B** and **Supplementary Table S3**). The number of non-overlapped sequence repeats varied from 28 in *P. dictyoneura* to 37 in *P. sinensis*. Among these non-overlapped repeats, palindromic repeats were the most common with 207, followed by forward repeats with 160; reverse repeats with 15 and complement repeats with 7 (**Supplementary Figure S2**). The repeat sequence analysis would provide help for the study of genetic variation in *Paraboea*.

### Comparative chloroplast genome analysis

**Figure 4:**
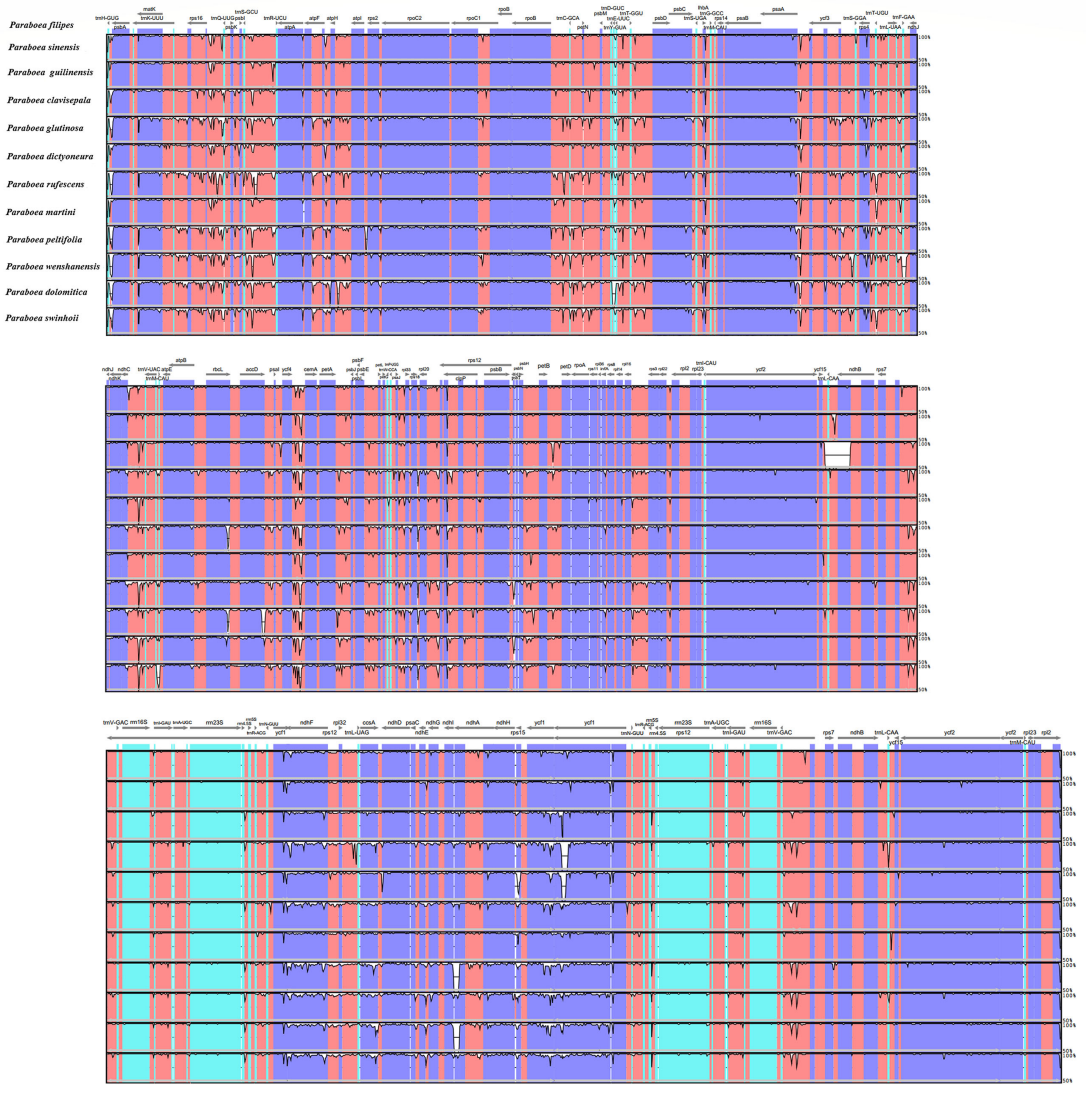
The comparative analysis with LAGAN program of the whole-chloroplast genome of twelve different species of *Paraboea*. The x-axis represents the coordinate in the chloroplast genome.

**Figure 5:**
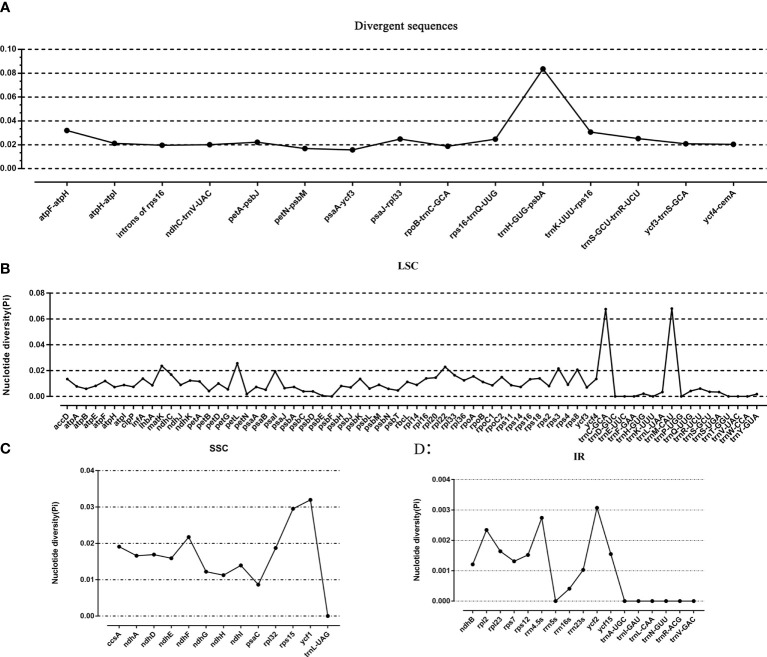
The nucleotide variability (Pi) value in the 12 *Paraboea* chloroplast genomes. **(A)** The Pi value of divergent sequences. **(B)** The Pi value of LSC region. **(C)** The Pi value of SSC region. **(D)** The Pi value of IR region.

Taking *P. sinensis* as a reference, multiple alignments of twelve *Paraboea* chloroplast genomes were conducted, and the results suggested that the non-coding sequences showed more divergence than the coding regions (**Figure 4**). According to the comparative analysis, the main divergent sequences for the noncoding regions were *atp*H-*atp*I, *atp*F-*atp*H, *rps16*-*trn*Q-UUG, *trn*K-UUU-*rps16*, *trn*H-GUG-*psb*A, *trn*S-GCU-*trn*R-UCU and *psa*A-*ycf3*, and the strongly divergent sequences for the coding regions were *mat*K, *pet*L, *ycf1*, *ycf2* and *ndh*F, which might be good candidates for *Paraboea* species identification. To quantify the levels of DNA polymorphism, we calculated the Pi values of above twelve regions, the Pi values of these regions were calculated ranged from 0.01569 (*psa*A-*ycf3*) to 0.08362 (*trn*H-GUG-*psb*A) (**Figure 5A**). The highest average Pi value of the coding regions was calculated in the SSC region, followed by the coding regions of the LSC and IR region (**Figures 5B–D**). The ten coding genes with the highest polymorphism in descending order include: *ycf1*, *rps15*, *pet*L, *mat*K, *rpl22*, *ndh*F, *rps3*, *rps8*, *psa*I and *ccs*A. The Pi values of tRNA and rRNA genes were also calculated, and the results showed that *trn*C-GCA and *trn*M-CAU had high Pi values, 0.0676 and 0.068, respectively. We finally screened out ten divergent regions with the highest value, which were *trn*H-GUG-*psb*A, *trn*M-CAU, *trn*C-GCA, *atp*F-*atp*H, *ycf1*, *trn*K-UUU-*rps16*, *rps15*, *pet*L, *trn*S-GCU-*trn*R-UCU and *psa*J-*rpl33*. These divergent regions may be the best candidate marker for DNA barcoding.

### Phylogenetic relationship

**Figure 6:**
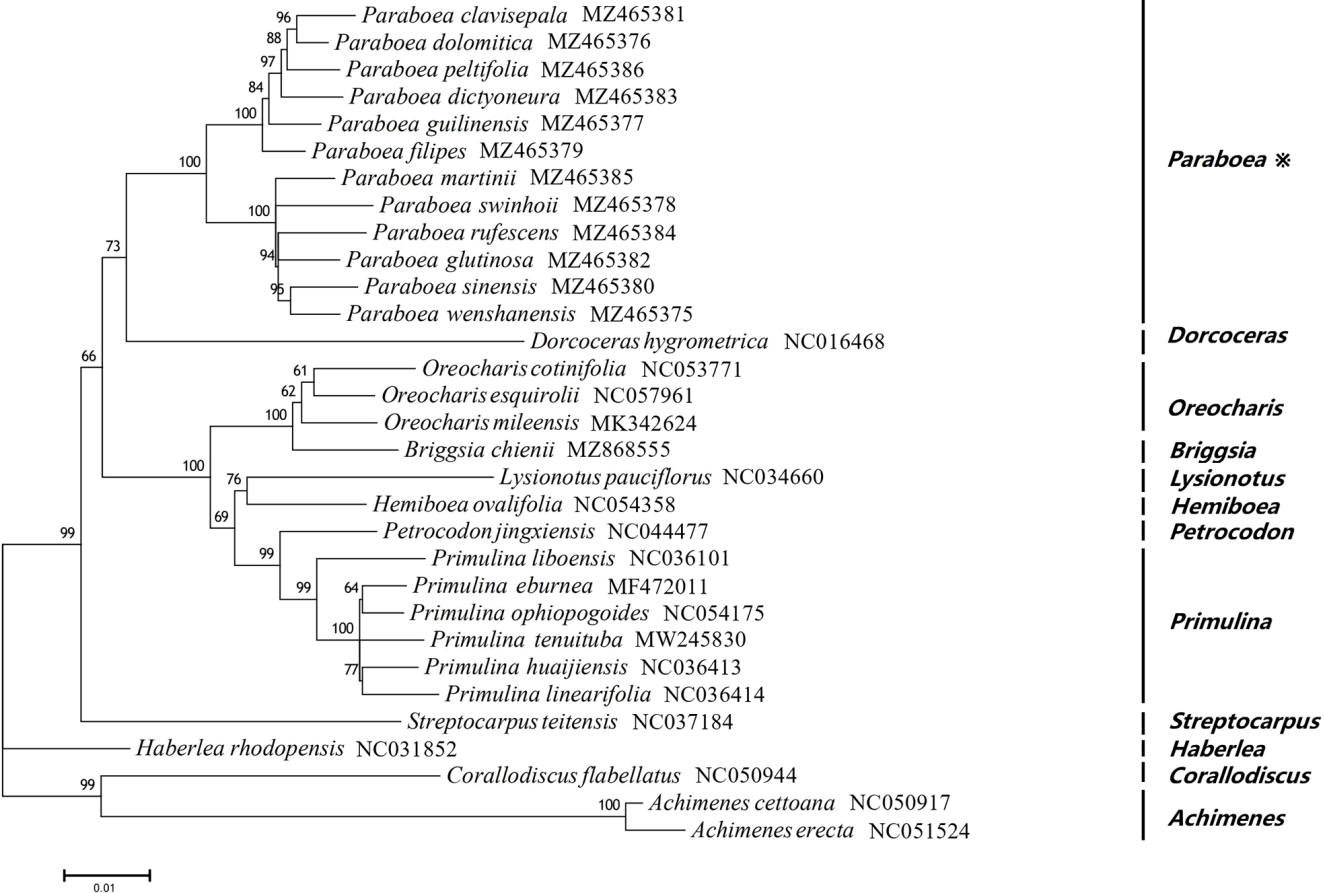
The ML phylogenetic tree of 31 Gesneriaceae species. Supporting values of > 50% for ML were shown on the branch.

In order to study the phylogenetic position of *Paraboea*, ML tree were constructed using 76 protein coding genes of the chloroplast genomes for 31 Gesneriaceae species, including 12 *Paraboea* species (**Figure 6**). Among the 31 Gesneriaceae species, except for 12 *Paraboea* species, the chloroplast genomes of the remaining species were obtained from NCBI (**Supplementary Table S1**). In the phylogenetic tree, all nodes were supported with bootstrap values greater than 60%, and each genus clustered together into a clade (100% bootstrap values). The 12 *Paraboea* species clustered into a clade, and then clustered with *Dorcoceras hygrometrica* (100% bootstrap values). *Paraboea* clade were divided into two major small clades with 100% bootstrap support value. In one major small clade, *P. clavisepala* and *P. dolomitica* form a clade, and then sequentially formed clades with *P. peltifolia*, *P. dictyoneura*, *P. guilinensis* and *P. filipes.* In another major small clade, the clade formed by *P. sinensis* and *P. wenshanensis*, clustered with *P. rufescens* and *P. glutinosa*, and then shared a sister relationship with *P. martinii* and *P. swinhoei.*

### Adaptive evolution analysis

**Figure 7:**
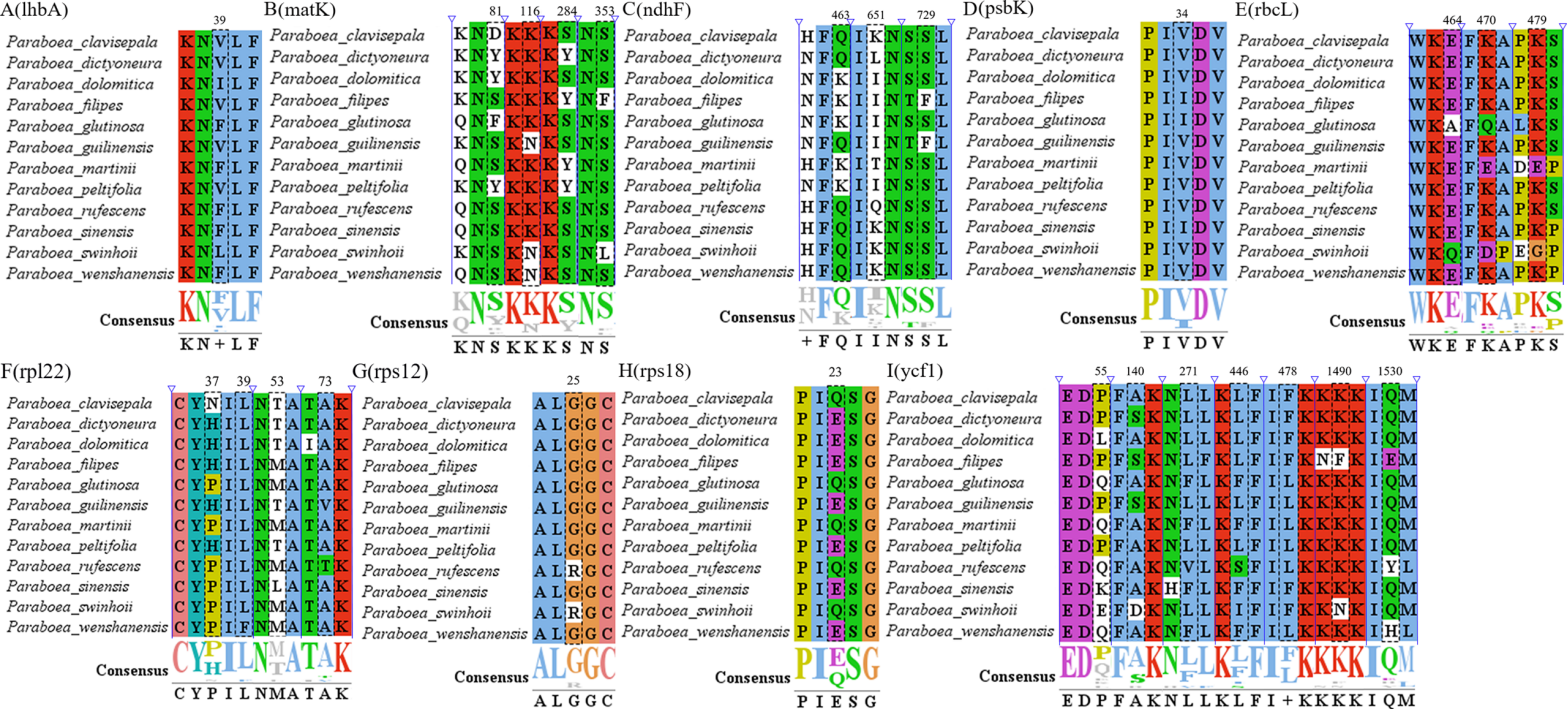
Nine genes of positive selection of amino acid sequences in site model tests.

**Figure 8:**
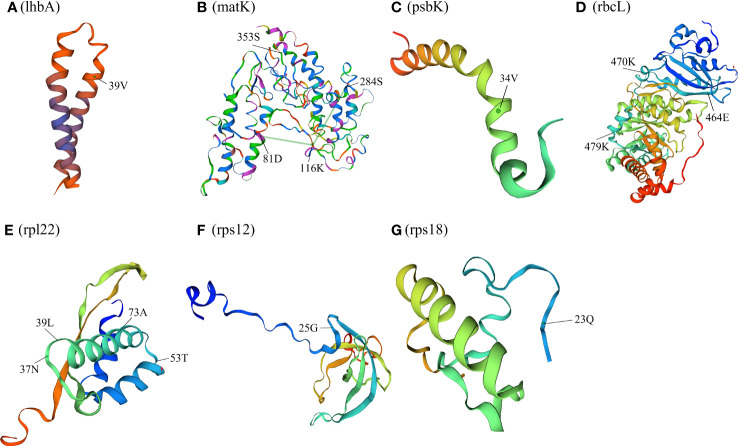
Spatial location of the positively selected sites in proteins of P. clavisepala. **(A)** Spatial location of the positively selected sites in the lhbA protein of P. clavisepala. **A, B** Spatial location of the positively selected sites in the matK protein of P. clavisepala. **A, C** Spatial location of the positively selected sites in the psbK protein of P. clavisepala. **A, D** Spatial location of the positively selected sites in the rbcL protein of P. clavisepala. **A, E** Spatial location of the positively selected sites in the rpl22 protein of P. clavisepala. **A, F** Spatial location of the positively selected sites in the rps12 protein of P. clavisepala. **A, G** Spatial location of the positively selected sites in the rps18 protein of P. clavisepala.

The 76 chloroplast protein coding genes of twelve *Paraboea* species were tested, and positive selection was found in nine genes (*lhb*A, *mat*K, *ndh*F, *psb*K, *rbc*L, *rpl22*, *rps12*, *rps18* and *ycf1*) with a high posterior probability (>95%) using the BEB test (**Figure 7** and **Supplementary Table S4**). One amino acid site (the 39th codon) was identified to be under positive selection in *lhb*A gene (**Figure 7A**). The spatial analysis of LhbA protein under positive selection indicated that the site was located in the α-helix (**Figure 8A**). Four amino acid sites (the 81th, 116th, 284th and 353th codons) under positive selection were detected in Maturase coded by *mat*K gene (**Figure 7B**). The spatial analysis indicated that two sites were located in α-helix, and the other sites were located at β-sheet and random coil, respectively (**Figure 8B**). In addition, three of nine genes were coding genes for photosynthesis: the *ndh*F gene for NADH dehydrogenase subunit F (NDHF), the *psb*K gene for Photosystem II subunits K (PsbK), and the *rbc*L gene for rubisco large subunit (RBCL). Three amino acid sites (463th, 651th and 729th) under positive selection in NDHF were located the random coil, α-helix and α-helix respectively (**Figure 7C** and **Supplementary Figure S3**). Based on the protein structure prediction, one amino acid site (34th) under positive selection in PsbK was located in α-helix (**Figure 7D** and **Figure 8C**). Three amino acid sites (464th, 470th and 479th) under positive selection in RBCL were located in the random coil (**Figure 7E** and **Figure 8D**).

Meanwhile, other three genes were coding genes for protein synthesis: *rps*12 and *rps*18 genes for Ribosomal protein smaller subunit (RPS), and *rpl*22 gene for Ribosomal protein larger subunit 22 (RPL22). One positive selection site was identified in RPS12 and RPS18 protein, respectively (**Figures 7G, H**; **Figures 8F, G**). Four positive selection sites were identified in RPL22 (**Figure 7F** and **Figure 8E**). Finally, seven sites were identified in YCF1 (Hypothetical chloroplast reading frame 1) coded by *ycf*1 gene (**Figure 7I** and **Supplementary Figure S4**). Based on the protein structure prediction, most of these positive selection sites were located in the α-helix, followed by random coil and β-sheet (**Figure 8**).

## Discussion

### Chloroplast genome features

In this study, the chloroplast genomes of twelve *Paraboea* species were characterized (**Figure 1**; **Table 2**). The twelve chloroplast genomes also showed a highly conserved feature in terms of structures, gene orders, gene numbers (protein-coding genes, rRNAs and tRNAs) and intron number. The chloroplast genomes of twelve *Paraboea* plants ranged from 153166 to 154245 bp in length. The chloroplast genomes of angiosperms have a highly conserved feature, but the contraction and expansion of the boundary between the IR and SC region is considered to be the main reason for the size change of the chloroplast genome (Zhang et al., 2016). The same phenomenon also existed in the twelve *Paraboea* chloroplast genomes. Despite the twelve *Paraboea* chloroplast genomes having well-conserved genomic structures including gene number and order, length variation of the whole sequences comprising IR, LSC and SSC regions was detected among these chloroplast genomes (**Figure 2**). In particular, *ycf1* and *ndh*F genes located at the SSC/IR border had the greatest variation in position and length in the twelve *Paraboea* chloroplast genomes. These sequence variations might be the result of boundary contraction and expansion between the SSC/IR regions in plants (Wang and Messing, 2011).

### Repeat sequence analysis

SSRs have been used as molecular markers for determining a high degree of variation in similar species and are helpful to explore population genetics and polymorphisms (Zhao et al., 2015). In total, 600 SSRs were detected in the twelve chloroplast genomes, 315 of which were mononucleotide repeats, accounting for the majority of all SSRs (52.50%) (**Figure 3A** and **Supplementary Figure S1**). Among the twelve chloroplast genomes, the number of mononucleotide repeats was the largest. In angiosperm chloroplast genomes, lots of similar results were also reported previously (Gandhi et al., 2010; Bessega et al., 2013). The results also demonstrated that the SSRs identified in the chloroplast genome were mostly made up of polyadenine (Poly-A) or polythymine (Poly-T) repeats, and the contents of guanine (Poly-G) and cytosine (Poly-C) repeats were low, which was consistent with the general SSR characteristics of chloroplast genomes in angiosperms (Ebert and Peakall, 2009; Asaf et al., 2020).

Moreover, 389 non-overlapped sequence repeats were identified in twelve chloroplast genomes (**Figure 3B** and **Supplementary Figure S2**), including the most non-overlapped sequence repeats (37) in *P. sinensis* and the least non-overlapped sequence repeats (28) in *P. dictyoneura*. Among the 389 repeats, there were four types: forward repetition, reverse repetition, complement repetition and palindrpmic repetition. Palindrpmic repetition and forward repetition accounted for the highest proportion. The same conclusion was obtained in the analysis of repetitive sequences in the chloroplast genomes of other Gesneriaceae plants (Gu et al., 2020). All of these SSRs, together with non-overlapped sequence repeats, are useful sources to develop markers for genetic diversity analysis of *Paraboea* species (**Supplementary Table S2** and **Supplementary Table S3**).

### Phylogenetic relationship

Most of the studies on the molecular phylogeny of *Paraboea* were based on chloroplast *trn*L-F sequences and nuclear ITS sequences (Puglisi et al., 2011; Xin et al., 2019; Guo et al., 2020). Strict consensus tree based on combined ITS and *trn*L-*trn*F sequences of 53 samples showed that *Paraboea* samples formed three major clades (Puglisi et al., 2011). The major clade 1 contains Chinese and Thai *Paraboea* species, some small clades of which were with low or no branch support. The existing two chloroplast sequences didn’t completely solve the phylogenetic relationship of *P. sinensis, P. rufescens, P. glanduliflora* and *P. swinhoei.*

In recent years, phylogeny based on the complete chloroplast genome has been widely used in plants (Feng et al., 2017; Kyalo et al., 2020; Tian and Wariss, 2021). 31species belonging to 12 genera of Gesneriaceae were used to construct the ML tree in this study. All nodes were supported with bootstrap values greater than 60%, and the 12 *Paraboea* species clustered into a clade with 100% bootstrap values (**Figure 6**), supporting the monophyly of *Paraboea*. The topology of the phylogenetic tree was more resolved than found in combined ITS and *trn*L-*trn*F datasets of previous studies (Puglisi et al., 2011; Xin et al., 2019; Guo et al., 2020). The clade formed by *P. sinensis* and *P. wenshanensis*, clustered with *P. rufescens* and *P. glutinosa*, and then shared a sister relationship with *P. martinii* and *P. swinhoei*. The sequences of chloroplast genome sequences could completely solve the phylogenetic relationship of *Paraboea*, and chloroplast genome data could provide more genetic information on the evolutionary relationships and phylogeny among species of Gesneriaceae.

### Adaptive evolution analysis

The plants of *Paraboea* are mainly distributed in karst areas of south and southwest China and southeast Asia. Karst is a unique and fragile ecological environment and the rocks forming karst landforms mainly consist of limestone, dolomite and other soluble carbonate rocks. (Li et al., 2019). Because of the thin soil layer, low water holding capacity and strong permeability of rocks, the stress of frequent alternation of dry and wet is common in karst habitats. Facing frequent temporary drought, karst plants generally appear enhanced photosynthetic capacity and light protection mechanisms (Liu et al., 2021). The changes in ground temperature, air temperature, light intensity and atmospheric relative humidity are quite different in different microhabitats (such as rocky hills with direct sunlight, forests with weak light and dark caves, etc.) (Ou et al., 2020). These challenging karst environments may impose selective pressure on genes, which could leave a footprint of natural selection in genes of chloroplast involved in adaptation to the environment. In this study, among the chloroplast genes of twelve *Paraboea* species, nine genes (*lhb*A, *mat*K, *ndh*F, *psb*K, *rbc*L, *rpl22*, *rps12*, *rps18* and *ycf1*) were identified under positive selection using a site model (**Figure 7**, **Figure 8** and **Supplementary Table S4**).

The maturase encoded by *mat*K gene is involved in splicing of introns of *trn*K, *trn*I, *atp*F and other genes, which is important for maintaining the normal function of chloroplast (Moran et al., 1994; Vogel et al., 1999; Lambowitz and Zimmerly, 2004; Stern et al., 2010; Zoschke et al., 2010). There were four positive selection sites in *mat*K gene of *Paraboea* species, and *mat*K gene also had undergone adaptive evolution in Lycopodiaceae, Bryophyta and other plants (Hao et al., 2010a; Hao et al., 2010b). Adaptive evolution of *mat*K may fine-tune its function to optimize its performance in various environmental conditions.

Three genes under positive selection were related to photosynthesis, namely *psb*K, *ndh*F and *rbc*L gene. The *psb*K gene encodes Photosystem II subunits K. Photosystem II is the first link in the chain of photosynthesis, and captures photons and uses the energy to extract electrons from water molecules (Ferreira et al., 2004). PSBK is not necessary for the assembly or activity of photosystem II complex, but is essential for optimal photosystem II function. The *psb*K gene was detected under positive selection in *Echinacanthus* (Acanthaceae) and *Calligonum Mongolicum* (Polygonaceae), and speculated to play an important role in plant adaptation evolutionary process to the diverse environment (Gao et al., 2019a; Duan et al., 2020). The *ndh*F gene encodes NADH dehydrogenase subunit protein (Kubicki et al., 1996). In previous studies on plant adaptive evolution, *ndh*F genes were often under positive selection pressure (Liu et al., 2020; Li et al., 2021; Wen et al., 2021). The NADH dehydrogenase complex of higher plants not only participated in photosynthetic electron transport (Joet et al., 2001; Joet, 2002), but also acted as an electron transport carrier for chloroplast respiration (Casano et al., 2000). The adaptive evolution of the *ndh*F gene may affect energy transformation and resistance to photooxidative stress in different environments. The *rbc*L gene encoded the gene coding for the rubisco large subunit protein of Rubisco, which was an important part of the photosynthesis electron transport regulator (Piot et al., 2018). The *rbc*L gene was often under positive selection because of being the target of selection diverse environment factors related to the changes in temperature, drought and carbon dioxide concentration (Fan et al., 2018). NADH-dehydrogenase subunits and Photosystem subunits were essential in the electron transport chain for the generation of ATP and light energy utilization, which were all indispensable parts for photosynthesis of plants (Yamori and Shikanai, 2016; Peltier et al., 2016). Therefore, the signature of positive selection in three genes related to photosynthesis suggests that they might have been involved in adaptation to diversified environments for *Paraboea* species in karst habitats.

Meanwhile, positive selection sites were also identified in *lhb*A, *rpl22*, *rps12* and *rps18* genes. The specific function of *lhb*A gene has not been fully studied (Wu et al., 2020). The *rps* genes encode small ribosomal subunit proteins, and *rpl* genes encode large ribosomal subunit proteins (Muto and Ushida, 1995). The mutation of genes encoded in ribosomal proteins under the pressure of the natural environment may affect the translation of chloroplast ribosome (Ramundo et al., 2013).

Seven sites were detected under positive selection in the *ycf1* gene. Positive selection of *ycf1* was also found to be involved in the adaptation of the genus *Panax* (Jiang et al., 2018). Being one of the largest chloroplast genes, the *ycf1* gene encoding a component of the chloroplast’s inner envelope membrane protein translocon, has become a useful gene for assessing sequence variations and evolutionary processes in plants (Huang et al., 2010; Kikuchi et al., 2013). The function and the adaptive evolutionary analysis of the *ycf1* gene would better understand the evolutionary mechanism of plants in the future.

Because of environmental pressure, adaptive evolution of chloroplast genomes is a common phenomenon, especially for genes involved in photosynthesis. Genes associated with photosynthesis are more likely to evolve adaptively in plants distributed in extreme environments, such as shade plants or aquatic plants (Xie et al., 2018). In karst areas, there are great differences in environmental factors such as light intensity, soil water content and nutrient availability, which might have exerted strong selective forces on plant evolution (Ai et al., 2015). In this study, nine chloroplast genes under positive selection, most of which were related to photosynthesis and protein synthesis, may possibly contribute to the diverse evolution and adaptation of *Paraboea* species to karst extreme environments.

## Conclusion

This is the first report of the complete chloroplast genome sequence of *Paraboea* species. In this study, the newly sequenced chloroplast genomes of twelve *Paraboea* species were reported and compared. The genome annotation and comparative analysis showed that each chloroplast genome was a typical quadripartite structure like traditional angiosperms, and the GC content, gene number and order were similar to each other. The chloroplast genomes of the twelve *Paraboea* species were similar in structure, composition and gene order. In the twelve *Paraboea* chloroplast genomes, a total of 600 SSRs and 389 non-overlapped sequence repeats were identified, which were informative sources for developing markers for genetic diversity analysis of *Paraboea* species. In addition, we found that 10 different regions (*trn*H-GUG-*psb*A, *trn*M-CAU, *trn*C-GCA, *atp*F-*atp*H, *ycf1*, *trn*K-UUU-*rps16*, *rps15*, *pet*L, *trn*S-GCU-*trn*R-UCU and *psa*J-*rpl33*) were potential molecular markers in twelve *Paraboea* species. The phylogenetic tree based on 76 protein coding genes clearly demonstrated the genetic and evolutionary relationships of 31 species belonging to 12 genera of Gesneriaceae. Adaptive evolution analysis detected positive selection signals in nine chloroplast genes (i.e., *lhb*A, *mat*K, *ndh*F, *psb*K, *rbc*L, *rpl22*, *rps12*, *rps18* and *ycf1*). The evolution of *Paraboea* to adapt to extreme habitats in karst environments may be linked to changes in these positive selection sites. These analyses of chloroplast genomes will provide preparations for the development and utilization of *Paraboea* species germplasm resources and the formulation of conservation strategies.

## Data availability statement

The datasets presented in this study can be found in online repositories. The names of the repository/repositories and accession number(s) can be found in the article/**Supplementary Material**.

## Author contributions

BZ, FW, YW, YM and XH conceived and designed the study. ZL, YW collected and analyzed the data. BZ, FW, YW, ZL and XH wrote the manuscript. All authors have directly contributed to this manuscript. All authors contributed to the article and approved the submitted version.

## Funding

This study is supported by the Key Sci. & Tech. Research and Development Project of Guangxi (Guike AD20159091 & ZY21195050), the Guangxi Natural Science Foundation (2020GXNSFBA297049), the capacity-building project of SBR of CAS (KFJ-BRP-017-68), the Anhui Provincial Natural Science Foundation (1908085QC1), the Fund of Yunnan Key Laboratory for Integrative Conservation of Plant Species with Extremely Small Populations (PSESP2021F07).

## Conflict of interest

The authors declare that the research was conducted in the absence of any commercial or financial relationships that could be construed as a potential conflict of interest.

## Publisher’s note

All claims expressed in this article are solely those of the authors and do not necessarily represent those of their affiliated organizations, or those of the publisher, the editors and the reviewers. Any product that may be evaluated in this article, or claim that may be made by its manufacturer, is not guaranteed or endorsed by the publisher.

## Supplementary material

Supplementary Figure 1 The comparison of each type of SSRs among 12 *Paraboea* choloplast genomes.

Supplementary Figure 2 Number of the four complex repeat types (forward, palindrome, reverse, and complement) in the twelve *Paraboea* chloroplast.

Supplementary Figure 3 Protein secondary structure of *ndh*F.

Supplementary Figure 4 Protein secondary structure of *ycf1*.

Supplementary Table 1 Chloroplast genome sequences from GenBank used in this study.

Supplementary Table 2 Summary of SSRs in twelve *Paraboea* chloroplast genomes.

Supplementary Table 3 Summary of complex repeats in twelve *Paraboea* chloroplast genomes.

Supplementary Table 4 Positive selection sites identified in the chloroplast genomes of twelve *Paraboea* species.

## References

[B1] Ai B. Gao Y. Zhang X. Tao J. Kang M. Huang H. (2015). Comparative transcriptome resources of eleven *Primulina* species, a group of ‘stone plants’ from a biodiversity hot spot. Mol. Ecol. Resour. 15, 619–632. doi: 10.1111/1755-0998.12333 25243665

[B2] Amiryousefi A. Hyvönen J. Poczai P. (2018). IRscope: an online program to visualize the junction sites of chloroplast genomes. Bioinformatics 34, 3030–3031. doi: 10.1093/bioinformatics/bty220 29659705

[B3] Asaf S. Jan R. Khan A. L. Lee I. J. (2020). Complete chloroplast genome characterization of *Oxalis corniculata* and its comparison with related species from family oxalidaceae. Plants 9, 928. doi: 10.3390/plants9080928 PMC7464629 32717796

[B4] Beier S. Thiel T. Munch T. Scholz U. Mascher M. (2017). MISA-web: a web server for microsatellite prediction. Bioinformatics 33 (16), 2583–2585. doi: 10.1093/bioinformatics/btx198 28398459 PMC5870701

[B5] Bessega C. F. Pometti C. L. Miller J. T. Watts R. Saidman B. O. Vilardi J. C. (2013). New microsatellite loci for *Prosopis alba* and *P. chilensis* (Fabaceae). Appl. Plant Sci. 1 (5), 1200324. doi: 10.3732/apps.1200324 PMC4105034 25202541

[B6] Buchan W. A. Minneci F. Nugent C. O. Bryson K. Jones D. T. (2013). Scalable web services for the PSIPRED protein analysis workbench. Nucleic Acids Res. 41, W349–W357. doi: 10.1093/nar/gkt381 23748958 PMC3692098

[B7] Casano L. M. Zapata J. M. Martin M. Sabater B. (2000). Chlororespiration and poising of cyclic electron transport. plastoquinone as electron transporter between thylakoid NADH dehydrogenase and peroxidase. J. Biol. Chem. 275 (2), 942–948. doi: 10.1074/jbc.275.2.942 10625631

[B8] Chen H. M. Chen Z. E. Du Q. Jiang M. Wang B. Liu C. (2022). Complete chloroplast genome of *Campsis grandiflora* (Thunb.) schum and systematic and comparative analysis within the family bignoniaceae. Mol. Biol. Rep. 49 (4), 3085–3098. doi: 10.1007/s11033-022-07139-0 35059974

[B9] Chen J. Zang Y. Shang S. Liang S. Zhu M. Wang Y.. (2021). Comparative chloroplast genomes of *Zosteraceae* species provide adaptive evolution insights into seagrass. Front. Plant Sci. 12. doi: 10.3389/fpls.2021.741152 PMC8495015 34630493

[B10] Dierckxsens N. Mardulyn P. Smits G. (2017). NOVOPlasty: *de novo* assembly of organelle genomes from whole genome data. Nucleic Acids Res. 45 (4), e18. doi: 10.1093/nar/gkw955 28204566 PMC5389512

[B11] Doyle J. J. Doyle J. L. (1987). A rapid DNA isolation procedure for small quantities of fresh leaf tissue. Phytochem. Bull. 19, 11–15.

[B12] Duan H. R. Zhang Q. Yang H. Tian F. P. Hu Y. Wang C. M.. (2020). Complete chloroplast genome of *Calligonum mongolicum*: Genome organization, codon usage pattern, phylogenetic relationships, comparative structure and adaptive evolution analysis. Research Square. doi: 10.21203/rs.3.rs-49271/v1

[B13] Ebert D. Peakall R. (2009). Chloroplast simple sequence repeats (cpSSRs): Technical resources and recommendations for expanding cpSSR discovery and applications to a wide array of plant species. Mol. Ecol. Res. 9, 673–690. doi: 10.1111/j.1755-0998.2008.02319.x 21564725

[B14] Edgar R. C. (2004). MUSCLE: multiple sequence alignment with high accuracy and high throughput. Nucleic Acids Res. 32 (5), 1792–1797. doi: 10.1093/nar/gkh340 15034147 PMC390337

[B15] Fan W. B. Wu Y. Yang J. Shahzad K. Li Z. H. (2018). Comparative chloroplast genomics of *Dipsacales* species: Insights into sequence variation, adaptive evolution and phylogenetic relationships. Front. Plant Sci. 9. doi: 10.3389/fpls.2018.00689 PMC5974163 29875791

[B16] Feng C. Xu M. Feng C. Kang M. (2017). The complete chloroplast genome of *Primulina* and two novel strategies for development of high polymorphic loci for population genetic and phylogenetic studies. BMC evol. Biol. 17 (1). doi: 10.3389/fpls.2018.00689 PMC5678776 29115917

[B17] Feng S. G. Zheng K. X. Jiao K. L. Cai Y. C. Chen C. L. Mao Y. Y.. (2020). Complete chloroplast genomes of four *Physalis* species (Solanaceae): lights into genome structure, comparative analysis, and phylogenetic relationships. BMC Plant Biol. 20 (1), 242. doi: 10.1186/s12870-020-02429-w 32466748 PMC7254759

[B18] Ferreira K. N. Iverson T. M. Maghlaoui K. Barber J. Iwata S. (2004). Architecture of the photosynthetic oxygen-evolving center. Science 303 (5665), 1831–1838. doi: 10.1126/science.1093087 14764885

[B19] Frazer K. A. Pachter L. Poliakov A. Rubin E. M. Dubchak I. (2004). VISTA: computational tools for comparative genomics. Nucleic Acids Res. 32, W273–W279. doi: 10.1093/nar/gkh458 15215394 PMC441596

[B20] Gandhi S. G. Awasthi P. Bedi Y. S. (2010). Analysis of SSR dynamics in chloroplast genomes of brassicaceae family. Bioinformation 5 (1), 16–20. doi: 10.6026/97320630005016 21346873 PMC3039999

[B21] Gao F. Chen C. Arab D. Du Z. He Y. Ho S. (2019b). EasyCodeML: a visual tool for analysis of selection using CodeML. Ecol. Evol. 9, 3891–3898. doi: 10.1002/ece3.5015 31015974 PMC6467853

[B22] Gao C. Deng Y. Wang J. (2019a). The complete chloroplast genomes of *Echinacanthus* species (Acanthaceae): Phylogenetic relationships, adaptive evolution, and screening of molecular markers. Front. Plant Sci. 9 1989. doi: 10.3389/fpls.2018.01989 30687376 PMC6335349

[B23] Gao J. Y. Ren P. Y. Yang Z. H. Li Q. J. (2006). The pollination ecology of *Paraboea rufescens* (Gesneriaceae): a buzz-pollinated tropical herb with mirror-image flowers. Ann. botany 97 (3), 371–376. doi: 10.1093/aob/mcj044 16371444 PMC2803634

[B24] Greiner S. Lehwark P. Bock R. (2019). OrganellarGenomeDRAW (OGDRAW) version 1.3.1: expanded toolkit for the graphical visualization of organellar genomes. Nucleic Acids Res. 47 (W1), W59–W64. doi: 10.1093/nar/gkz238 30949694 PMC6602502

[B25] Gu C. H. Dong B. Xu L. Tembrock L. R. Zheng S. Y. Wu Z. Q. (2018). The complete chloroplast genome of *H. myrtifolia* and comparative analysis within myrtales. Molecules1019831 23, 846. doi: 10.3390/molecules23040846 PMC6017443 29642470

[B26] Guo J. Meng T. Pang H. Zhang Q. (2016). *Petrocodon retroflexus* sp. nov. (gesneriaceae) from a karst cave in guizhou, China. Nordic J. Botany 34 (2), 159–164. doi: 10.1111/njb.00941

[B27] Guo Z. Wu Z. Xu W. Li Z. Xiang X. (2020). *Paraboea dolomitica* (Gesneriaceae), a new species from guizhou, China. PhytoKeys 153, 37–48. doi: 10.3897/phytokeys.153.50933 32765179 PMC7381718

[B28] Gu L. Su T. An M. T. Hu G. X. (2020). The complete chloroplast genome of the vulnerable *Oreocharis esquirolii* (Gesneriaceae): Structural features, comparative and phylogenetic analysis. Plants (Basel) 9 (12), 1692. doi: 10.3390/plants9121692 PMC7760870 33276435

[B29] Hao D. C. Chen S. L. Xiao P. G. (2010a). Molecular evolution and positive Darwinian selection of the chloroplast maturase *mat*K. J. Plant Res. 123 (2), 241–247. doi: 10.1007/s10265-009-0261-5 19943076

[B30] Hao D. C. Mu J. Chen S. L. Xiao P. G. (2010b). Physicochemical evolution and positive selection of the gymnosperm *mat*K proteins. J. Genet. 89 (1), 81–89. doi: 10.1007/s12041-010-0014-1 20505250

[B31] Huang J. L. Sun GL Zhang D. M. (2010). Molecular evolution and phylogeny of the angiosperm *ycf2* gene. J. Syst. Evol. 48, 240–248. doi: 10.1111/j.1759-6831.2010.00080.x

[B32] Huelsenbeck J. Ronquist F. (2001). MRBAYES: Bayesian inference of phylogenetic trees. Bioinformatics 17, 754–755. doi: 10.1093/bioinformatics/17.8.754 11524383

[B33] Jiang P. Shi F. X. Li M. R. Liu B. Wen J. Xiao H. X.. (2018). Positive selection driving cytoplasmic genome evolution of the medicinally important ginseng plant genus *Panax* . Front. Plant sci. 9. doi: 10.3389/fpls.2018.00359 PMC5893753 29670636

[B34] Joet T. (2002). Cyclic electron flow around photosystem I in C3 plants. *In vivo* control by the redox state of chloroplasts and involvement of the NADH-dehydrogenase complex. Plant Physiol. 128 (2), 760–769. doi: 10.1104/pp.010775 11842179 PMC148937

[B35] Joet T. CournAc L. Horvath E. M. Peltier M. G. (2001). Increased sensitivity of photosynthesis to antimycin induced by inactivation of the chloroplast ndhB gene. evidence for a participation of the NADH-dehydrogenase complex to cyclic electron flow around photosystem I. Plant Physiol. 125 (4), 1919–1929. doi: 10.1104/pp.125.4.1919 11299371 PMC88847

[B36] Kazutaka K. Standley D. M. (2013). MAFFT multiple sequence alignment software version 7: improvements in performance and usability. Mol. Biol. Evol. 30, 772–780. doi: 10.1093/molbev/mst010 23329690 PMC3603318

[B37] Kikuchi S. Bédard J. Hirano M. Hirabayashi Y. Oishi M. Imai M.. (2013). Uncovering the protein translocon at the chloroplast inner envelope membrane. Science 339, 571–574. doi: 10.1126/science.1229262 23372012

[B38] Kubicki A. Funk E. Steinmüller W. K. (1996). Differential expression of plastome-encoded ndh genes in mesophyll and bundle-sheath chloroplasts of the C4plant sorghum bicolor indicates that the complex I-homologous NAD(P)H-plastoquinone oxidoreductase is involved in cyclic electron transport. Planta 199 (2), 276–281. doi: 10.1002/anie.200905829

[B39] Kurtz S. Choudhuri J. V. Ohlebusch E. Schleiermacher C. Stoye J. Giegerich R. (2001). REPuter: the manifold applications of repeat analysis on a genomic scale. Nucleic Acids Res. 29, 4633–4642. doi: 10.1093/nar/29.22.4633 11713313 PMC92531

[B40] Kyalo C. M. Li Z. Z. Mkala E. M. Malombe I. Hu G. W. Wang Q. F. (2020). The first glimpse of *Streptocarpus ionanthus* (Gesneriaceae) phylogenomics: Analysis of five subspecies' chloroplast genomes. Plants 9 (4), 456. doi: 10.3390/plants9040456 PMC7238178 32260377

[B41] Lambowitz A. M. Zimmerly S. (2004). Mobile group II introns. Annu. Rev. Genet. 38, 1–35. doi: 10.1146/annurev.genet.38.072902.091600 15568970

[B42] Langmead B. Trapnell C. Pop M. Salzberg S. L. (2009). Ultrafast and memory-efficient alignment of short DNA sequences to the human genome. Genome Biol. 10 (3), R25. doi: 10.1186/gb-2009-10-3-r25 19261174 PMC2690996

[B43] Li F. He X. Sun Y. Zhang X. Tang X. Li Y.. (2019). Distinct endophytes are used by diverse plants for adaptation to karst regions. Sci. Rep. 9 (1), 5246. doi: 10.1038/s41598-019-41802-0 30918319 PMC6437200

[B44] Li J. L. Tang J. M. Zeng S. Y. Han F. Yuan J. Yu J. (2021). Comparative plastid genomics of four *Pilea* (Urticaceae) species: insight into interspecific plastid genome diversity in pilea. BMC Plant Biol. 21 (1), 25. doi: 10.1186/s12870-020-02793-7 33413130 PMC7792329

[B45] Liu C. Huang Y. Wu F. Liu W. Ning Y. Huang Z.. (2021). Plant adaptability in karst regions. J. Plant Res. 134 (5), 889–906. doi: 10.1007/s10265-021-01330-3 34258691

[B46] Liu Q. Li X. Li M. Xu W. Heslop-Harrison J. S. (2020). Comparative chloroplast genome analyses of *Avena*: insights into evolutionary dynamics and phylogeny. BMC Plant Biol. 20 (1), 406. doi: 10.1186/s12870-020-02621-y 32878602 PMC7466839

[B47] Li J. M. Wang Y. Z. (2007). Phylogenetic reconstruction among species of *Chiritopsis* and *Chirita sect. gibbosaccus* (Gesneriaceae) based on nrDNA ITS and cpDNA trnL-f sequences. Syst. Botany 32 (4), 888–898. doi: 10.1600/036364407783390764

[B48] Moran J. V. Mecklenburg K. L. Sass P. Belcher S. M. Mahnke D. Lewin A.. (1994). Splicing defective mutants of the COXI gene of yeast mitochondrial DNA: initial definition of the maturase domain of the group II intron AI2. Nucleic Acids Res. 22 (11), 2057–2064. doi: 10.1093/nar/22.11.2057 8029012 PMC308121

[B49] Muto A. Ushida C. (1995). Transcription and translation. Methods Cell Biol. 48, 483. doi: 10.1186/s12870-020-02621-y 8531739

[B50] Ou Z. Pang S. He Q. Peng Y. Huang X. Shen W. (2020). Effects of vegetation restoration and environmental factors on understory vascular plants in a typical karst ecosystem in southern China. Sci. Rep. 10 (1), 1–10. doi: 10.1038/s41598-020-68785-7 32694713 PMC7374739

[B51] Peltier G. Aro E. M. Shikanai T. (2016). NDH-1 and NDH-2 plastoquinone reductases in oxygenic photosynthesis. Annu. Rev. Plant Biol. 67, 55–80. doi: 10.1146/annurev-arplant-043014-114752 26735062

[B52] Piot A. Hackel J. Christin P. A. Besnard G. (2018). One-third of the plastid genes evolved under positive selection in PACMAD grasses. Planta 247, 255–266. doi: 10.1007/s00425-017-2781-x 28956160

[B53] Puglisi C. Middleton D. J. Triboun P. Möller M. (2011). New insights into the relationships between *Paraboea*, *Trisepalum* and *Phylloboea* (Gesneriaceae) and their taxonomic consequences. Taxon 60 (6), 1693–1702. doi: 10.1002/tax.606014

[B54] Puglisi C. Phutthai T. (2017). A new species of *Paraboea* (Gesneriaceae) from Thailand. Edinburgh J. Botany 75 (1), 51–54. doi: 10.1017/S0960428617000324

[B55] Ramundo S. Rahire M. Schaad O. Rochaix J. D. (2013). Repression of essential chloroplast genes reveals new signaling pathways and regulatory feedback loops in *Chlamydomonas* . Plant Cell. 25, 167–186. doi: 10.1105/tpc.112.103051 23292734 PMC3584532

[B56] Rozas J. Ferrer-Mata A. Sánchez-DelBarrio J. C. Guirao-Rico S. Librado P. Ramos-Onsins S. E.. (2017). DnaSP 6: DNA sequence polymorphism analysis of large data sets. Mol. Biol. Evol. 34 (12), 3299–3302. doi: 10.1093/molbev/msx248 29029172

[B57] Schattner P. Brooks A. N. Lowe T. M. (2005). The tRNAscan-SE, snoscan and snoGPS web servers for the detection of tRNAs and snoRNAs. Nucleic Acids Res. 33, W686–W689. doi: 10.1093/nar/gki366 15980563 PMC1160127

[B58] Shi L. Chen H. Jiang M. Wang L. Wu X. Huang L.. (2019). CPGAVAS2, an integrated plastome sequence annotator and analyzer. Nucleic Acids Res. 47 (W1), W65–w73. doi: 10.1093/nar/gkz345 31066451 PMC6602467

[B59] Song Y. Zhao W. J. Xu J. Li M. F. Zhang Y. J. (2022). Chloroplast genome evolution and species identification of *Styrax* (Styracaceae). BioMed. Res. Int. 22, 5364094. doi: 10.1155/2022/5364094 PMC8893999 35252450

[B60] Stamatakis A. (2006). RAxML-VI-HPC: maximum likelihood-based phylogenetic analyses with thousands of taxa and mixed models. Bioinformatics 22, 2688–2690. doi: 10.1093/bioinformatics/btl446 16928733

[B61] Stern D. B. Goldschmidt-Clermont M. Hanson M. R. (2010). Chloroplast RNA metabolism. Annu. Rev. Plant Biol. 61 (1), 125–155. doi: 10.1146/annurev-arplant-042809-112242 20192740

[B62] Tamura K. Stecher G. Peterson D. Filipski A. Kumar S. (2013). MEGA6: molecular evolutionary genetics analysis version 6.0. Mol. Biol. Evol. 30, 2725–2729. doi: 10.1093/molbev/mst197 24132122 PMC3840312

[B63] Tao J. Feng C. Ai B. Kang M. (2016). Adaptive molecular evolution of the two-pore channel 1 gene TPC1 in the karst-adapted genus *Primulina* (Gesneriaceae). Ann. botany 118 (7), 1257–1268. doi: 10.1093/aob/mcw168 PMC5155596 27582362

[B64] Tian X. Wariss H. M. (2021). The complete chloroplast genome sequence of *Metabriggsia ovalifolia* w. t. Wang (Gesneriaceae), a national key protected plant endemic to karst areas in China. Mitochondrial DNA B Resour. 6 (3), 833–834. doi: 10.1080/23802359.2021.1884021 33763595 PMC7954431

[B65] Vogel J. Borner T. Hess W. R. (1999). Comparative analysis of splicing of the complete set of chloroplast group II introns in three higher plant mutants. Nucleic Acids Res. 27 (19), 3866–3874. doi: 10.1093/nar/27.19.3866 10481026 PMC148650

[B66] Wang W. Messing J. (2011). High-throughput sequencing of three lemnoideae (duckweeds) chloroplast genomes from. PloS One 6, e24670. doi: 10.1371/journal.pone.0024670 21931804 PMC3170387

[B67] Waterhouse A. Bertoni M. Bienert S. Studer G. Tauriello G. Gumienny R.. (2018). SWISS- MODEL: homology modelling of protein structures and complexes. Nucleic Acids Res. 46, W296–W303. doi: 10.1093/nar/gky427 29788355 PMC6030848

[B68] Wen F. Hong X. Chen L. Y. Zhou S. B. Wei Y. G. (2013). A new species of *Paraboea* (Gesneriaceae) from a karst limestone hill in southwestern guangdong, China. Phytotaxa 131 (1), 1–8. doi: 10.11646/phytotaxa.131.1.1

[B69] Wen F. Wu X. Li T. Jia M. Liu X. Liao L. (2021). The complete chloroplast genome of *Stauntonia chinensis* and compared analysis revealed adaptive evolution of subfamily lardizabaloideae species in China. BMC Genomics 22 (1), 161. doi: 10.1186/s12864-021-07484-7 33676415 PMC7937279

[B70] Wu Z. H. Liao R. Yang T. G. Dong X. Lan D. Q. Qin R.. (2020). Analysis of six chloroplast genomes provides insight into the evolution of *Chrysosplenium* (Saxifragaceae). BMC Genomics 21 (1), 621. doi: 10.1186/s12864-020-07045-4 32912155 PMC7488271

[B71] Xie D. F. Yu Y. Deng Y. Q. Li J. Liu H. Y. Zhou S. D.. (2018). Comparative analysis of the chloroplast genomes of the Chinese endemic genus *Urophysa* and their contribution to chloroplast phylogeny and adaptive evolution. Int. J. Mol. Sci. 19 (7), 1847. doi: 10.3390/ijms19071847 PMC6073864 29932433

[B72] Xin Z. B. Chou W. C. Maciejewski S. Fu L. F. Wen F. (2021). *Primulina papillosa* (Gesneriaceae), a new species from limestone areas of guangxi, China. PhytoKeys 177, 55–61. doi: 10.3897/arphapreprints.e63933 34025141 PMC8134418

[B73] Xin Z. B. Fu L. F. Fu Z. X. Li S. Wei Y. G. Wen F.. (2019). Complete chloroplast genome sequence of *Petrocodon jingxiensis* (Gesneriaceae). Mitochondrial DNA Part B. 4 (2), 2771–2772. doi: 10.1080/23802359.2019.1624208 33365721 PMC7687431

[B74] Xu W. B. Huang Y. S. Wei G. F. Tan W. N. Liu Y. (2012). *Paraboea angustifolia* (Gesneriaceae): A new species from limestone areas in northern guangxi, China. Phytotaxa 62 (1), 39–43. doi: 10.1007/s12228-010-9175-8

[B75] Yamori W. Shikanai T. (2016). Physiological functions of cyclic electron transport around photosystem I in sustaining photosynthesis and plant growth. Annu. Rev. Plant Biol. 67, 81–106. doi: 10.1146/annurev-arplant-043015-112002 26927905

[B76] Yang X. Xie D. F. Chen J. P. Zhou S. D. Yu Y. He X. J. (2020). Comparative analysis of the complete chloroplast genomes in *Allium* subgenus *Cyathophora* (Amaryllidaceae): Phylogenetic relationship and adaptive evolution. BioMed. Res. Int. 20, 1732586. doi: 10.1155/2020/1732586 PMC7201574 32420321

[B77] Zhai Y. F. Yu X. Q. Zhou J. G. Li J. Tian Z. Wang P. Q.. (2021). Complete chloroplast genome sequencing and comparative analysis reveals changes to the chloroplast genome after allopolyploidization in *Cucumis* . Genome 64 (6), 627–638. doi: 10.1139/gen-2020-0134 33460340

[B78] Zhang Y. J. Du L. W. Liu A. Chen J. J. Wu L. Hu W. M.. (2016). The complete chloroplast genome sequences of five *Epimedium* species: lights into phylogenetic and taxonomic analyses. Front. Plant Sci. 7. doi: 10.3389/fpls.2016.00306 PMC4791396 27014326

[B79] Zhang R. Zhang L. Wang W. Zhang Z. Du H. Qu Z.. (2018). Differences in codon usage bias between photosynthesis-related genes and genetic system-related genes of chloroplast genomes in cultivated and wild *Solanum* species. Int. J. Mol. Sci. 19 (10), 3142. doi: 10.3390/ijms19103142 PMC6213243 30322061

[B80] Zhao Y. B. Yin J. L. Guo H. Y. Zhang Y. Y. Xiao W. Sun C.. (2015). The complete chloroplast genome provides insight into the evolution and polymorphism of *Panax ginseng* . Front. Plant Sci. 5. doi: 10.3389/fpls.2014.00696 PMC4294130 25642231

[B81] Zhou P. Gu Z. J. Möller M. (2003). New chromosome counts and nuclear characteristics for some members of gesneriaceae subfamily cyrtandroideae from China and Vietnam. Edinburgh J. Botany 60 (3), 449–466. doi: 10.1017/S0960428603000349

[B82] Zoschke R. Nakamura M. Liere K. Sugiura M. Börner T. Schmitz-Linneweber C.. (2010). An organellar maturase associates with multiple group II introns. Proc. Natl. Acad. Sci. U. S. A. 107 (7), 3245–3250. doi: 10.1073/pnas.0909400107 20133623 PMC2840290

