# Supplementary figures and images for: Comparative chloroplast genome analyses of Paraboea (Gesneriaceae): Insights into adaptive evolution and phylogenetic analysis
Source: Front Plant Sci. 2022 Oct 5;13:1019831. doi: 10.3389/fpls.2022.1019831 (PMC9581172; doi:10.3389/fpls.2022.1019831)

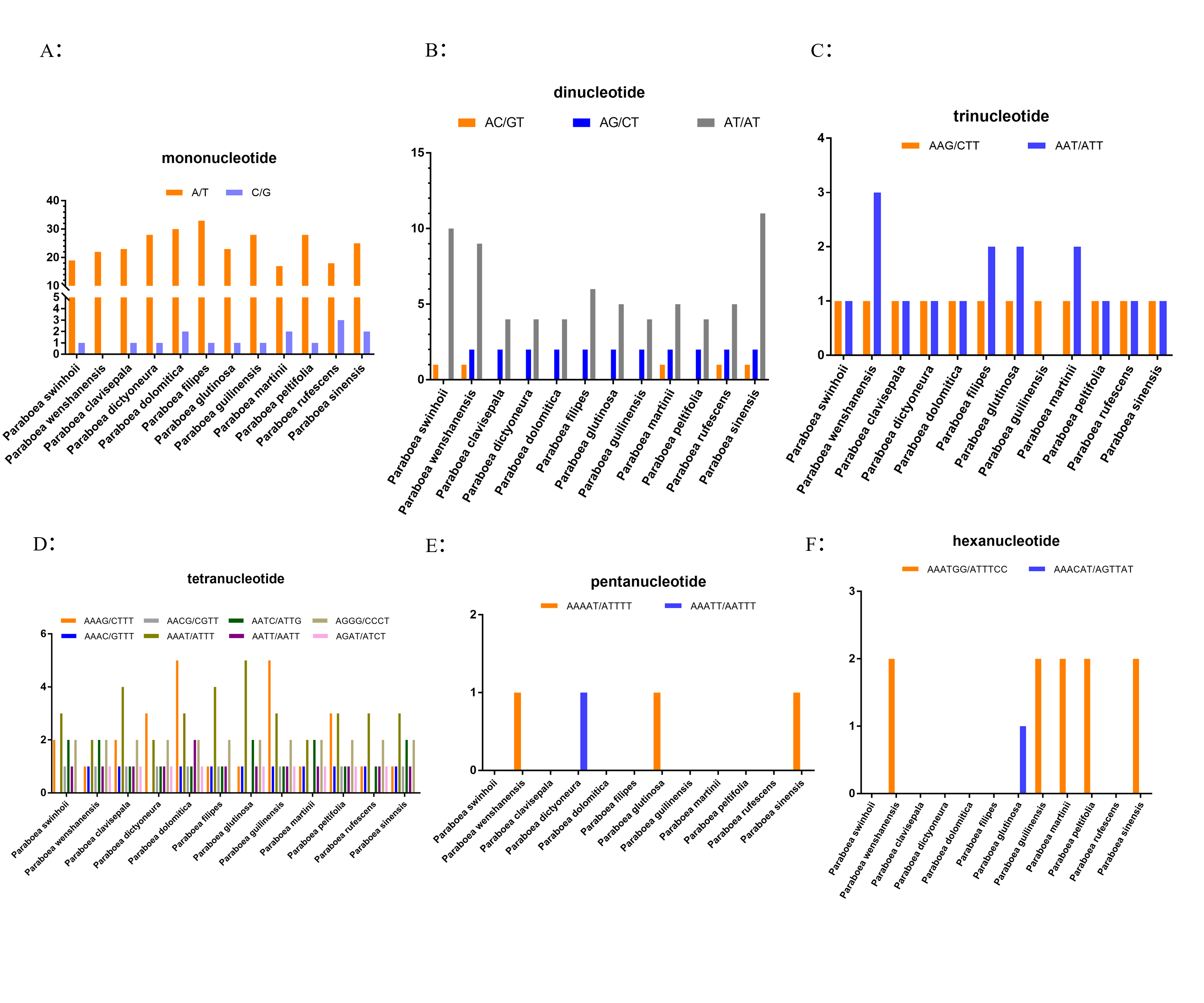

Supplement: Supplementary Figure 1 — The comparison of each type of SSRs among 12 Paraboea choloplast genomes. [file Image_1.tif]

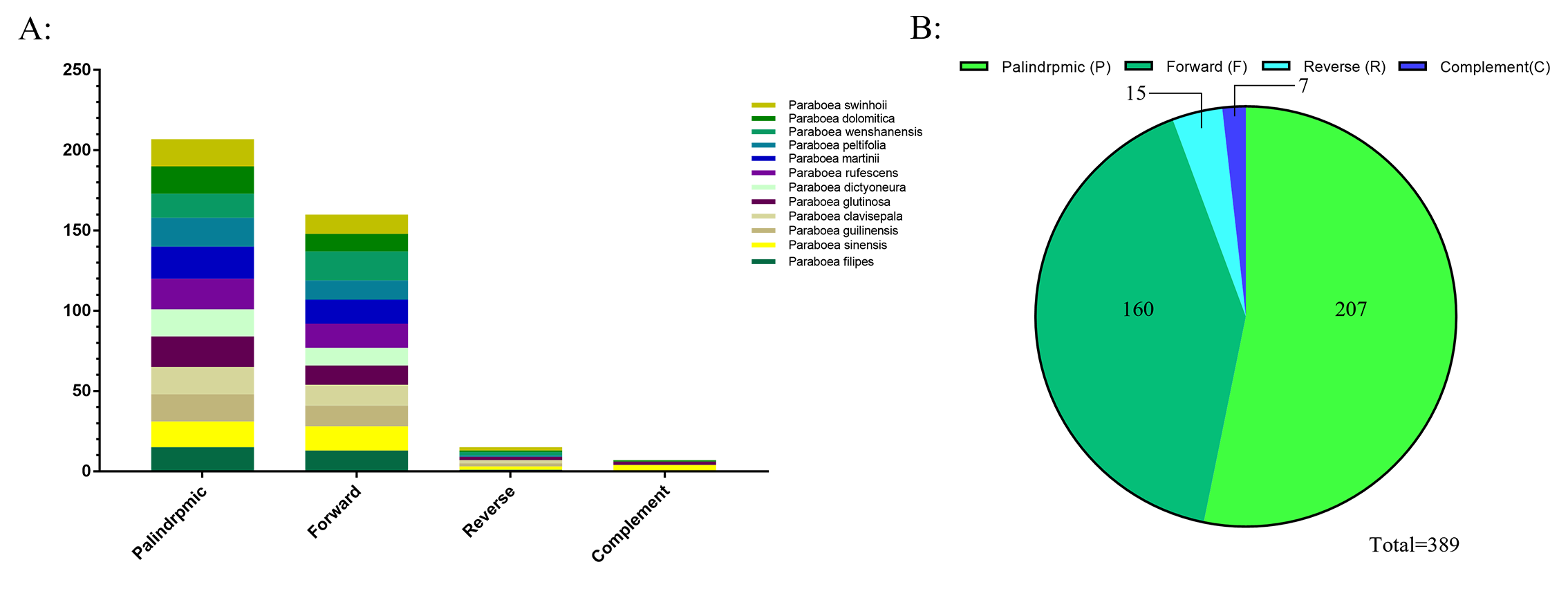

Supplement: Supplementary Figure 2 — Number of the four complex repeat types (forward, palindrome, reverse, and complement) in the twelve Paraboea chloroplast. [file Image_2.tif]

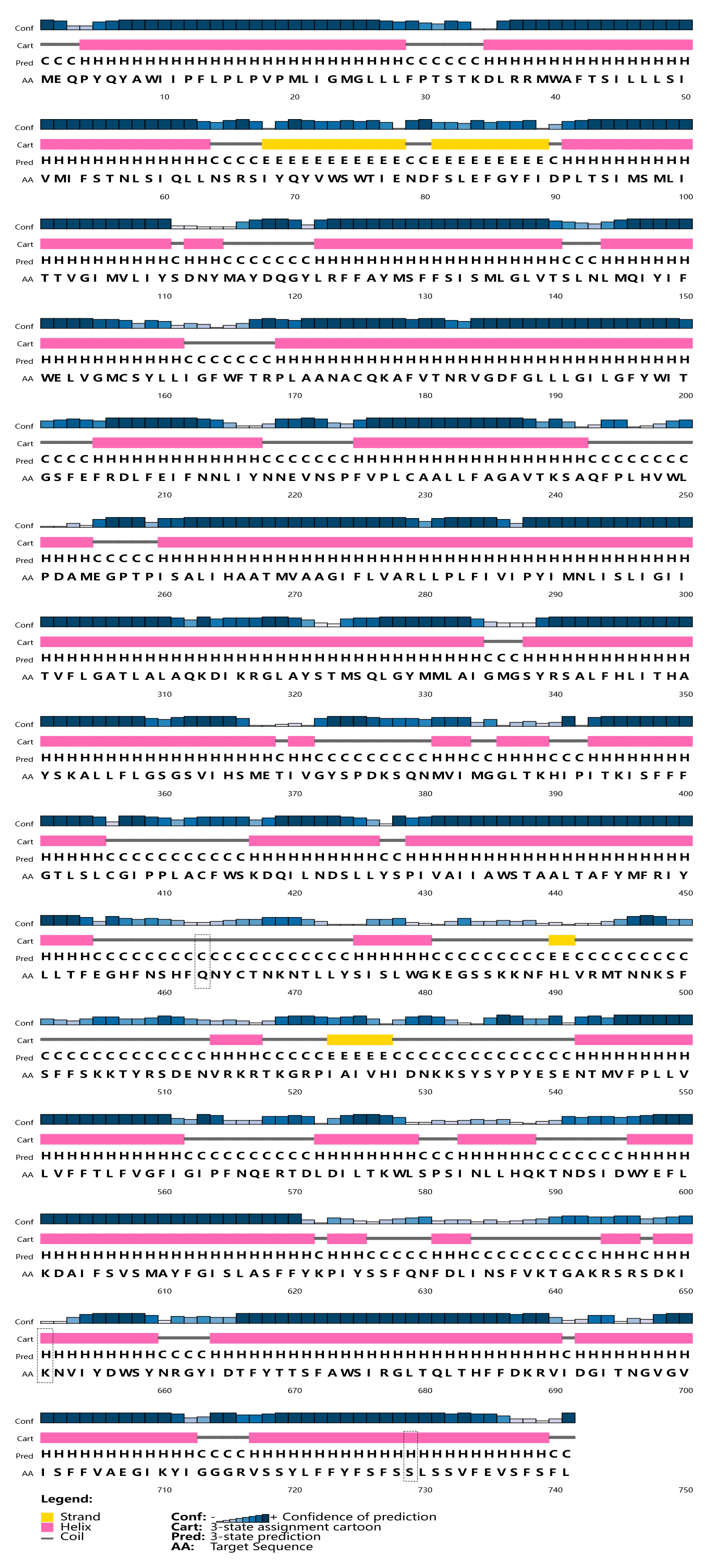

Supplement: Supplementary Figure 3 — Protein secondary structure of ndhF. [file Image_3.tif]

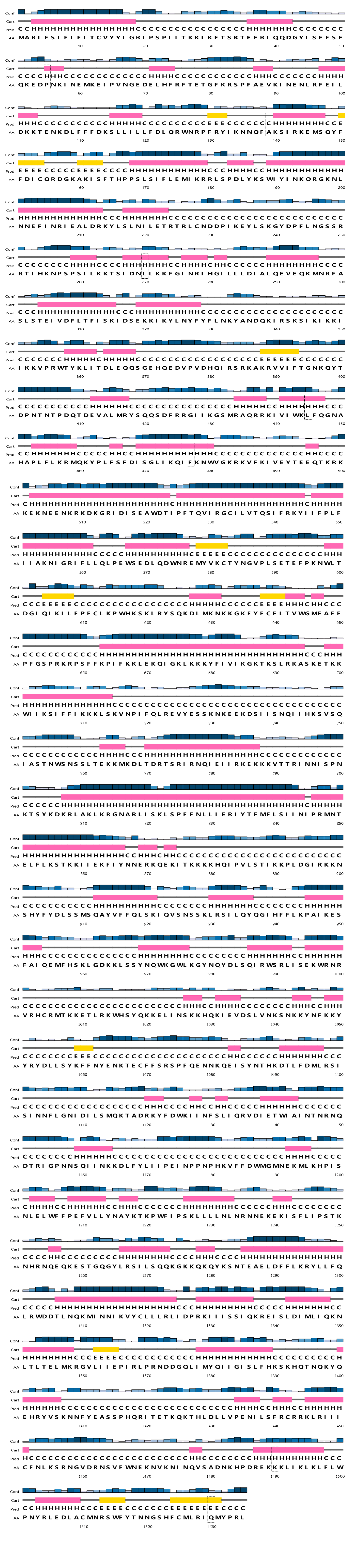

Supplement: Supplementary Figure 4 — Protein secondary structure of ycf1. [file Image_4.tif]
