# Supplementary material for: Comparative chloroplast genome analyses of Paraboea (Gesneriaceae): Insights into adaptive evolution and phylogenetic analysis
Source: Front Plant Sci. 2022 Oct 5;13:1019831. doi: 10.3389/fpls.2022.1019831 (PMC9581172; doi:10.3389/fpls.2022.1019831)
Supplement: Supplementary Table 1 — Chloroplast genome sequences from GenBank used in this study. [file DataSheet_1.pdf]

Supplementary Table S1 Chloroplast genome sequences from GenBank used in this study.

Species	Genbank accession numbers
Achimenes cettoana	NC050917
Achimenes erecta	NC051524
Briggsia chienii	MZ868555
Corallodiscus flabellatus	NC050944
Dorcoceras hygrometrica	NC016468
Hemiboea ovalifolia	NC054358
Haberlea rhodopensis	NC031852
Lysionotus pauciflorus	NC034660
Oreocharis cotinifolia	NC053771
Oreocharis esquirolii	NC057961
Oreocharis mileensis	MK342624
Petrocodon jingxiensis	NC044477
Primulina eburnea	MF472011
Primulina huaijiensis	NC036413
Primulina liboensis	NC036101
Primulina linearifolia	NC036414
Primulina ophiopogoides	NC054175
Primulina tenuituba	MW245830
Streptocarpus teitensis	NC037184

Supplementary Table S2 Summary of SSRs in twelve *Paraboea* chloroplast genomes.

	**Mono**	**Di**	**Tri**	**Tetra**	**Penta**	**Hexa**	**Total**
P. clavisepala	24	6	2	13	0	0	45
P. dictyoneura	29	6	2	11	1	0	49
P. dolomitica	32	6	2	16	0	0	56
P. filipes	34	8	3	11	0	0	56
P. glutinosa	29	6	1	15	2	0	53
P. guilinensis	29	6	1	15	2	0	53
P. martinii	19	8	3	10	2	0	42
P. peltifolia	29	6	2	13	2	0	52
P. rufescens	21	8	2	10	0	0	41
P. sinensis	27	14	2	11	1	2	57
P. swinhoei	20	11	2	11	0	0	44
P. wenshanensis	22	12	4	11	1	2	52
Total	315	97	26	147	11	4	600

Supplementary Table S3 Summary of complex repeats in twelve *Paraboea* chloroplast genomes.

	**Palindrpmic(P)**	**Forward(F)**	**Reverse(R)**	**Complement(C)**	**Total**
P. clavisepala	17	13	2	0	32
P. dictyoneura	17	11	0	0	28
P. dolomitica	17	11	1	1	30
P. filipes	15	13	1	0	29
P. glutinosa	19	12	2	2	35
P. guilinensis	17	13	2	0	32
P. martinii	20	15	0	0	35
P. peltifolia	18	12	1	0	31
P. rufescens	19	15	0	0	34
P. sinensis	16	15	2	4	37
P. swinhoei	17	12	2	0	31
P. wenshanensis	15	18	2	0	35
Total	207	160	15	7	389

Supplementary Table S4 Positive selection sites identified in the chloroplast genomes of twelve *Paraboea* species.

Category	Gene group	Gene	Model	LnL	LRT P-value	Positive sites
Other gene	LhbA	*lhbA*	M3	-289.524394	M0 vs. M3: 0.000561125	39 V 0 .999**
			M0	-292.203041	M1a vs. M2a: 0.000894753	
			M2a	-289.524394	M7 vs.M8: 0.000031185	
			M1a	-291.550145	M8a vs.M8: 0.046417792	
			M8	-289.566960		
			M7	-291.694490		
			M8a	-291.550144		
Photosynthesis	Photosystem II	*psbK*	M3	-307.633201	M0 vs. M3: 0.013648395	37 V 0.991**
			M0	-313.912348	M1a vs. M2a: 0.054446702	
			M2a	-307.695728	M7 vs.M8: 0.017704897	
			M1a	-310.606261	M8a vs.M8: 0.025643525	
			M8	-308.116306		
			M7	-310.671143		
			M8a	-310.606254		
	Rubisco large subunit	*rbcL*	M3	-2483.516379	M0 vs. M3: 0.000251488	464 E 0.967*
			M0	-2526.772549	M1a vs. M2a: 0.000014224	470 K 0.996**
			M2a	-2484.195903	M7 vs.M8: 0.001489651	479 K 0.954*
			M1a	-2495.356476	M8a vs.M8: 0.000003806	
			M8	-2484.676516		
			M7	-2508.777891		
			M8a	-2495.356531		

Category	Gene group	Gene	Model	LnL	LRT P-value	Positive sites
	NADH dehydrogenase	*ndhF*	M3	-918.406064	M0 vs. M3: 0.029409458	463 Q 0.990*
			M0	-923.785598	M1a vs. M2a: 0.092942524	651 K 0.986*
			M2a	-918.420150	M7 vs.M8: 0.009761691	729 S 0.992**
			M1a	-920.795924	M8a vs.M8: 0.028903700	
			M8	-918.410454		
			M7	-920.821051		
			M8a	-920.797124		
Protein synthesis	Ribosomal protein genes (larger subunit)	*rpl22*	M3	-666.526300	M0 vs. M3: 0.000109044	37 N 0.993**
			M0	-678.188700	M1a vs. M2a: 0.020746805	39 L 0.956*
			M2a	-666.526300	M7 vs.M8: 0.006084054	53 T 0.994**
			M1a	-670.401663	M8a vs.M8: 0.005411205	73 A 0.959*
			M8	-666.533353		
			M7	-671.635437		
			M8a	-670.401662		
		rps12	M3	-490.815627	M0 vs. M3: 0.000057005	25 G 0.967*
			M0	-503.180636	M1a vs. M2a: 0.000050387	
			M2a	-490.815627	M7 vs.M8: 0.000026379	
			M1a	-500.711397	M8a vs.M8: 0.000008636	
			M8	-490.815623		
			M7	-501.358575		
			M8a	-500.711387		

Category	Gene group	Gene	Model	LnL	LRT P-value	Positive sites
	Ribosomal protein genes (smaller subunit)	*rps18*	M3	-510.574703	M0 vs. M3: 0.014692531	23 Q 0.991**
			M0	-516.768312	M1a vs. M2a: 0.001504091	
			M2a	-510.574703	M7 vs.M8: 0.013451465	
			M1a	-512.768397	M8a vs.M8: 0.038129499	
			M8	-510.618771		
			M7	-513.376251		
			M8a	-512.768389		
Miscellaneous group	Maturase	*matK*	M3	-3283.900309	M0 vs. M3: 0.000000261	81 D 0.982*
			M0	-3302.007205	M1a vs. M2a: 0.000566563	116 K 0.951*
			M2a	-3283.901124	M7 vs.M8: 0.000123130	284 S 0.991**
			M1a	-3291.377046	M8a vs.M8: 0.000121152	353 S 0.966*
			M8	-3283.989717		
			M7	-3292.991983		
			M8a	-3291.377052		
Pseudogene unknown function	Hypothetical chloroplast reading frames (ycf)	*ycf1*	M3	-10683.325702	M0 vs. M3: 0.000462159	55 P 0.992**
			M0	-10801.125395	M1a vs. M2a: 0.000621935	140 A 0.987*
			M2a	-10686.398692	M7 vs.M8: 0.000771632	271 L 0.955*
			M1a	-10747.971742	M8a vs.M8: 0.000162958	446 L 0.973*
			M8	-10687.892099		478 F 0.999**
			M7	-10749.413224		1490 K 0.991**
			M8a	-10747.986327		1530 Q 0.997**

"*" Indicate that the posterior probabilities of the site are > 0.95.

"**" Indicate that the posterior probabilities of the site are > 0.99.
